# Articular cartilage repair biomaterials: strategies and applications

**DOI:** 10.1016/j.mtbio.2024.100948

**Published:** 2024-01-06

**Authors:** Mingkai Wang, Yan Wu, Guangfeng Li, Qiushui Lin, Wencai Zhang, Han Liu, Jiacan Su

**Affiliations:** a Institute of Translational Medicine, Shanghai University, Shanghai, 200444, China; b Organoid Research Center, Shanghai University, Shanghai, 200444, China; c College of Medicine, Shanghai University, Shanghai, 200444, China; d Department of Orthopedics Trauma, Shanghai Zhongye Hospital, Shanghai, 200941, China; e Department of Spine Surgery, The First Affiliated Hospital of Naval Medical University, Shanghai, 200433, China; f Department of Orthopedics, The First Affiliated Hospital Jinan University, Guangzhou, 510632, China; g Department of Orthopedics, Xinhua Hospital, Shanghai Jiao Tong University School of Medicine, Shanghai, 200092, China

**Keywords:** Cartilage, Repair strategies, Mechanically supported scaffolds, Biologically active substances, Clinical practice

## Abstract

Articular cartilage injury is a frequent worldwide disease, while effective treatment is urgently needed. Due to lack of blood vessels and nerves, the ability of cartilage to self-repair is limited. Despite the availability of various clinical treatments, unfavorable prognoses and complications remain prevalent. However, the advent of tissue engineering and regenerative medicine has generated considerable interests in using biomaterials for articular cartilage repair. Nevertheless, there remains a notable scarcity of comprehensive reviews that provide an in-depth exploration of the various strategies and applications. Herein, we present an overview of the primary biomaterials and bioactive substances from the tissue engineering perspective to repair articular cartilage. The strategies include regeneration, substitution, and immunization. We comprehensively delineate the influence of mechanically supportive scaffolds on cellular behavior, shedding light on emerging scaffold technologies, including stimuli-responsive smart scaffolds, 3D-printed scaffolds, and cartilage bionic scaffolds. Biologically active substances, including bioactive factors, stem cells, extracellular vesicles (EVs), and cartilage organoids, are elucidated for their roles in regulating the activity of chondrocytes. Furthermore, the composite bioactive scaffolds produced industrially to put into clinical use, are also explicitly presented. This review offers innovative solutions for treating articular cartilage ailments and emphasizes the potential of biomaterials for articular cartilage repair in clinical translation.

## Graphical abstract

**Figure:**
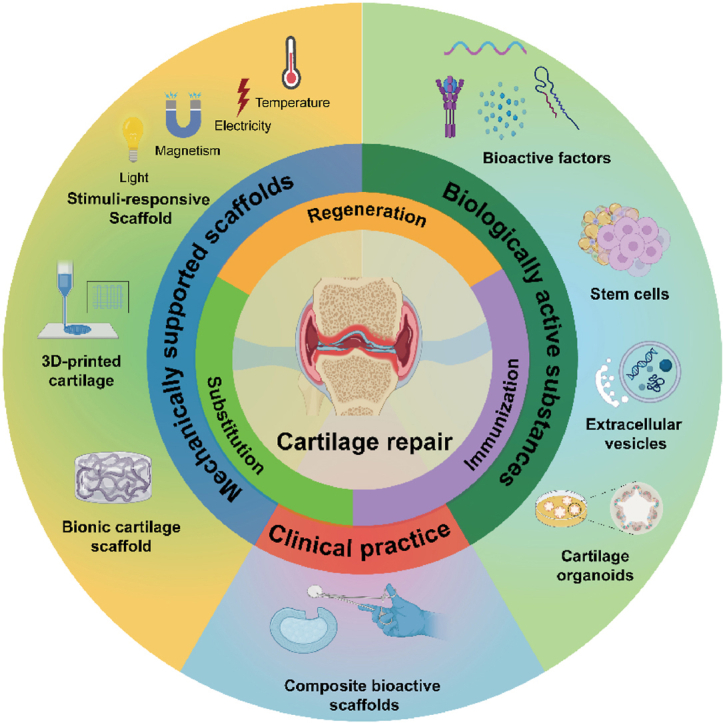

## 1 Introduction

Articular cartilage injury has emerged as a prevalent global health issue in recent years, posing a significant treatment challenge in orthopedics and sports medicine [1]. In a study involving 1000 patients undergoing knee arthroscopy, 61 % exhibited cartilage or osteochondral pathology signs. Additionally, 19 % of patients displayed focal cartilage or osteochondral defects [2]. Articular cartilage is a smooth, elastic, and translucent connective tissue that bears loads and reduces joint friction [3]. In cases of cartilage injury, the first structural damage occurs in the cartilage surface layer, leading to the loss of proteoglycans in the extracellular matrix (ECM) and disrupting the collagen fiber network [4]. Subsequently, the chondrocytes degenerate and are lost, resulting in minor localized damage that may spread to the middle and deep layers of cartilage [5]. Cartilage tissue has few cells, no blood vessels, lymph, or nerves, which restricts the ability to repair after injury [6]. Furthermore, poor cartilage damage treatment can cause degenerative arthritis, meniscus injury, bone hyperplasia, and other joint diseases [7,8].

Several clinical techniques are available for treating cartilage injuries, including microfracture technology, osteochondral transplantation technology [9,10], autologous chondrocyte transplantation technology and matrix-induced chondrogenesis technology [11]. However, these techniques have significant limitations, such as the challenge of repairing large areas of injury, the immune response of the patient, and the limited availability of donor tissue [12]. In arthroscopic techniques, progenitor cells are recruited from the bloodstream and bone marrow into the voids created by microdrilling or microfracture [13]. While this approach promotes cartilage regeneration by inducing differentiation of these cells into chondrogenic phenotypes, it results in mechanically weak cartilage that often degenerates into severe osteoarthritis later on [14]. Transplanting soft tissues like perichondrium and periosteum to full-thickness articular cartilage defects can lead to graft calcification, inadequate attachment to the defects, and high rates of graft loss [15,16]. Several non-surgical approaches are employed to control disease progression, such as oral nonsteroidal anti-inflammatory drugs, intra-articular hyaluronic acid injections, and platelet-rich plasma injections. Although clinical outcomes demonstrate certain efficacy in early pain relief and cartilage nourishment, the inherent characteristics of cartilage limit the effectiveness of conservative treatments [17]. Currently, the restoration of damaged cartilage and the deceleration of joint cartilage degeneration remain significant challenges in the clinical setting. Therefore, it is imperative to develop biomaterials that effectively promote physiological cartilage regeneration and repair to overcome the limitations of current clinical techniques.

**Fig. 1:**
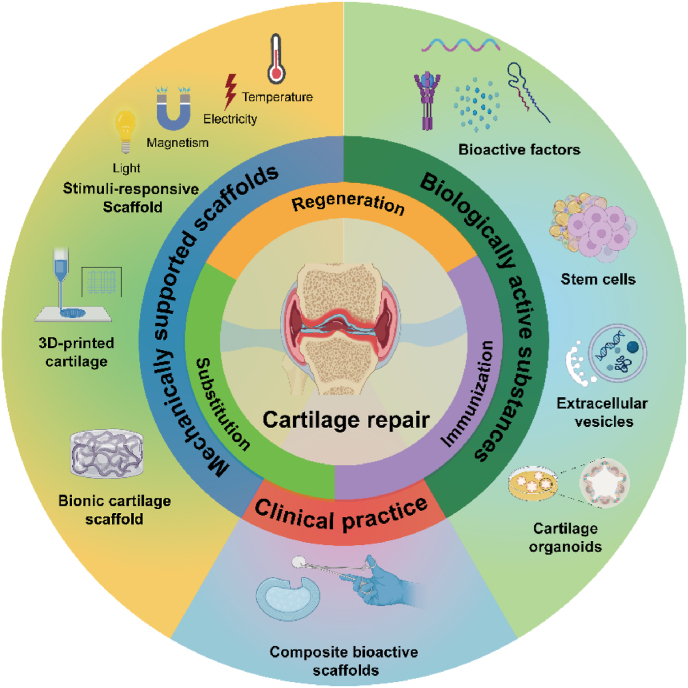
Schematic diagram of three cartilage repair strategies including regeneration, replacement, and immunization, as well as mechanical support of scaffolds, bioactive substances, and their clinical application. The figure was created with https://app.biorender.com/.

The emergence of regenerative medicine and tissue engineering has brought about more opportunities for cartilage repair. Given the mechanical properties, specific shape, and biological activity of cartilage, repair strategies can be tailored to suit different properties [18]. Researchers have made significant strides in developing biomaterials that address clinical and technical challenges [[19], [20], [21]]. This review provides a comprehensive overview of the latest strategies for cartilage repair, categorized into three types: regeneration, substitution, and immunization. Stimuli-responsive smart scaffold can accurately detect and treat cartilage damage by specifically responding to physical signals. The emerging scaffolds provide adequate mechanical support and tenacity for chondrocytes and the cell interstitium, such as 3D-printed and cartilage bionic scaffolds. Biologically active substances can accurately repair cartilage function, mainly promoting chondrocyte proliferation and differentiation by bioactive factors, stem cells, EVs, and organoids. Further, various commercial products are produced to treat cartilage injury (Fig. 1). This review offers insights into the mechanisms of cartilage injury repair and their potential clinical significance.

## 2 Articular cartilage repair strategies

### 2.1 Regeneration

**Fig. 2:**
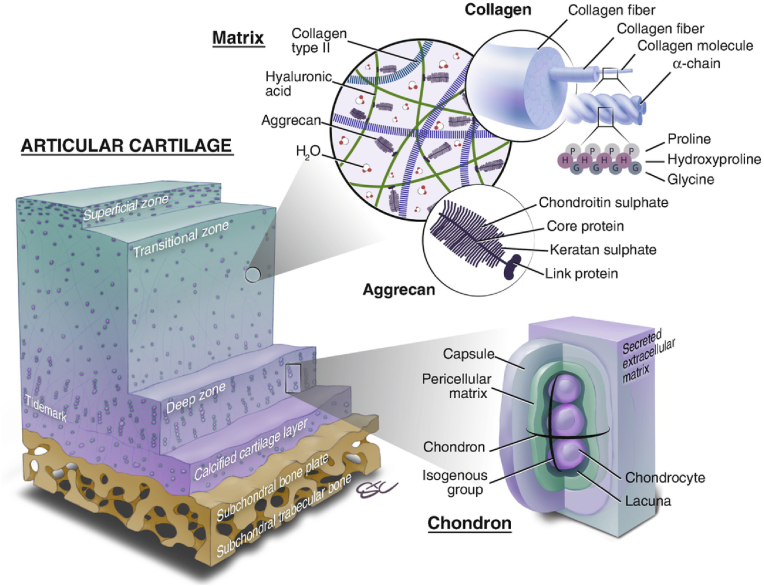
The architecture of osteochondral units and the clarification of their constituents. Reproduced with permission [25]. Copyright 2019, Springer Nature.

In natural articular cartilage, typically measuring 2–4 mm in thickness, the primary constituents are highly specialized chondrocytes and a dense ECM [22]. Chondrocytes exhibit reduced synthetic metabolism and proliferation rates, primarily relying on glycolysis for energy production. Therefore, effective cartilage regeneration strategies necessitate the stimulation of chondrocyte proliferation and stem cell differentiation [23]. Recent advancements in techniques such as 3D printing, microfluidics, and multiphase composite scaffolds have significantly advanced the regeneration of both bone and cartilage tissues. Successful strategies for regenerating bone-cartilage structures aim to restore structural, biomechanical, and biochemical characteristics consistent with native tissue [24]. Tissue engineering approaches, which incorporate scaffolds, cells, and bioactive factors, address the requirements for both structural reconstruction and tissue vitality (Fig. 2).

Initially, scaffold-free approaches involving the injection of stem cells, *N*-acetyl-d-glucosamine, and growth factors into cartilage-defective regions were explored [26]. Despite their cost-effectiveness and reduced surgical complexity, these strategies often yield suboptimal outcomes by failing to fully restore cartilage smoothness and integrity, leading to ongoing cartilage tissue damage. Moreover, injected substances encounter challenges in effectively penetrating the injured area [27]. To address these challenges, researchers have devised scaffold-based techniques that offer a matrix guiding therapeutic agents to deeper tissue layers [28]. Central to cartilage regeneration are seed cells, with chondrocytes being the primary choice, as studies suggest that implanting chondrocytes can lead to the regeneration of more functional cartilage tissue [29]. Stem cells, with their clonogenic potential, self-renewal capacity, and controllable differentiation capabilities, provide an alternative to chondrocytes [30]. The two main cell lineages employed for cartilage repair are bone marrow-derived mesenchymal stem cells (BMSCs) and adipose-derived mesenchymal stem cells (ADSCs), though a consensus on the superior cell type remains elusive [31].

Novel cartilage-inductive factors, including small molecules and peptides, have demonstrated heightened efficacy in promoting cartilage formation [32]. Additionally, mesenchymal stem cell-secreted paracrine factors have been recognized for facilitating cartilage tissue regeneration. Key factors such as the transforming growth factor (TGF), bone morphogenetic protein, and insulin-like growth factor (IGF) contribute to improved cell organization within the cartilage layer and stimulate new bone-cartilage regeneration [33]. Extracellular vesicles, encapsulating miRNA, proteins, and other bioactive molecules that enhance cartilage regeneration, have garnered significant attention [34]. Notably, hypoxia plays a crucial role in driving mesenchymal stem cell-mediated cartilage formation [35]. The hypoxic microenvironment within joint cavities, alongside hypoxia-inducible factors such as hypoxia-inducible factor-1α (HIF-1α), are pivotal contributors to the initiation of MSC-driven cartilage formation. HIF promotes cartilage formation by upregulating Sox9 expression, primarily by inhibiting peroxisome proliferator-activated receptor gamma-2 activation via the PI3K/Akt/FoxO pathway, thereby suppressing adipogenesis [36].

Current objectives in cartilage regeneration surpass mere tissue formation, emphasizing the attainment of cartilage regeneration under pathological conditions, mirroring clinical complexities [37]. Addressing full-thickness bone-cartilage defects, growth plate deficiencies, and load-bearing cartilage injuries presents formidable challenges in reestablishing cartilage morphology and functionality [38]. A prevalent strategy involves prioritizing the restoration of tissue morphology akin to the host tissue at precise locations and tailoring scaffolds to replicate the native tissue structure at distinct injury sites.

### 2.2 Substitution

In severe articular cartilage defects, cartilage substitution can address regeneration limitations [39]. Natural joint cartilage-bone tissue encompasses distinct regions, including the non-calcified cartilage layer, cartilage calcified layer, and subchondral bone layer [40]. Chondrocytes express cartilage-specific markers like Sox9 in the superficial cartilage layer, contributing to articulation lubrication [41]. In contrast, the calcified cartilage layer features elevated glycosaminoglycan and type II collagen levels (Col II) alongside inorganic components like poorly crystalline hydroxyapatite, crucial for stress transmission and substance exchange between cartilage and bone [42]. Within the subchondral bone region, osteocytes prominently express alkaline phosphatase and osteocalcin, providing mechanical support to the cartilage-bone interface. It is imperative to advance cartilage biomimetic materials to enhance cartilage repair materials based on existing technology and research findings [43]. Enhancing reparative mechanisms in articular cartilage injuries necessitates the development of multi-layered or gradient materials encompassing the superficial cartilage layer, calcified cartilage region, and subchondral bone domain. Cartilage substitution involves using materials and techniques that emulate biological systems' properties and functions to repair or regenerate damaged cartilage tissue [44]. Rooted in the principles of biomimetics, this approach aims to create biomaterials replicating cartilage's architecture and function [45,46]. Thus, ongoing efforts should focus on improving clinical cartilage repair materials and exploring new technologies.

Biomimetic cartilage materials hold a central role in joint replacement, striving to replicate natural cartilage properties to restore joint function, alleviate pain, and enhance the stability of compromised joints [47]. Their development and clinical application demand a multifaceted approach, including criteria such as (1) biocompatibility free from toxic effects or immune reactions, (2) effective integration at the biointerface, (3) robust performance in the human microenvironment, resisting degradation, electrolysis, and corrosion, (4) suitability for mass production, and (5) biomechanical compatibility with the surrounding tissues at the implantation site. Current materials predominantly comprise rigid plastics and metals. For example, the cartilage substitute Cartiva®SCI employs polyethylene glycol colloid [48]. These existing implants encounter poor integration, microplastic generation, and tissue compatibility issues. Therefore, the imperative lies in developing innovative synthetic cartilage substitutes that faithfully replicate the inherent characteristics of natural cartilage.

### 2.3 Immunization

Cartilage defects are frequently linked to inflammation, particularly in osteoarthritis (OA) and rheumatoid arthritis (RA) [49]. Nevertheless, achieving optimal cartilage regeneration necessitates effective control of inflammatory diseases. OA is characterized by irreversible cartilage degradation, where matrix metalloproteinases (MMPs) are recognized as critical pathogenic factors, accelerating the breakdown of cartilage matrix [50]. The introduction of biologics such as PRP and exosomes has demonstrated efficacy in downregulating MMPs [51]. Clinical trials involving targeted drugs, including recombinant IL-1 receptor antagonists (e.g., anakinra) and monoclonal antibodies against TNF-α (e.g., infliximab and adalimumab), offer promising prospects for reinstating a cartilage-friendly microenvironment while addressing RA [52].

**Fig. 3:**
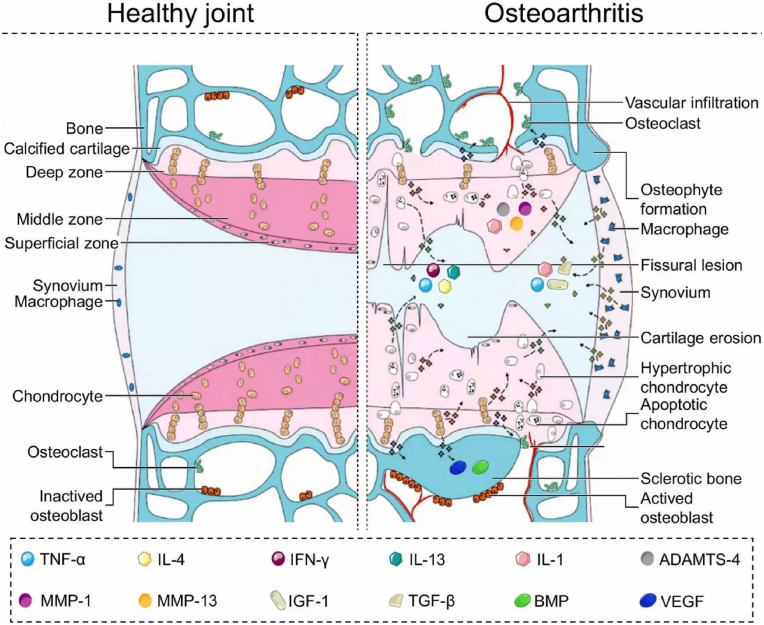
Signaling pathways and cartilage structural changes in the development of osteoarthritis. Reproduced with permission Reproduced with permission [54]. Copyright 2022, Elsevier.

Cartilage tissue regeneration represents a dynamic equilibrium encompassing cellular metabolism, differentiation, migration, and intricate interactions between the immune and musculoskeletal systems [24]. Inflammation serves a dual function in maintaining tissue homeostasis; it acts as a protective response to facilitate tissue regeneration while often being the primary driver of tissue damage in infectious diseases, immune disorders, and trauma [53]. Following an injury-induced inflammatory response, a specialized immune microenvironment directs the repair sequence of cells derived from peripheral blood mononuclear cells. This immune microenvironment plays a pivotal role in tissue healing, repair, and regeneration, and can be influenced by intrinsic and extrinsic factors, such as stem cells (Fig. 3).

A variety of bioactive cytokines can regulate the immune microenvironment, including interleukin (IL)-4, IL-10, interferon-γ (IFN-γ), and prostaglandin E2 [55]. Diverse tissue-specific biomaterials, designed with cytokine and immune-regulatory properties, are under development to augment tissue regeneration at sites of damage [53]. These biomaterial-based approaches offer structural support for transplanted cells, promoting their rapid proliferation and differentiation to replace lost tissue cells. Furthermore, biomaterials can enhance treatment efficacy when they possess immune-regulatory capabilities and mitigate locally overactive immune responses [56]. The attributes of such biomaterials vary based on the specific damaged tissue type, and their immune-suppressive properties play a pivotal role in shaping tissue repair and regeneration outcomes.

## 3 Mechanically supported scaffolds

Cartilage is crucial in providing mechanical support, absorbing pressure and shock, dispersing pressure, buffering shock absorption, and reducing joint friction [57]. Due to its unique physical properties, cartilage can maintain structural integrity even under heavy loads [58]. However, cartilage damage resulting from trauma or osteoarthritis requires biomaterials with mechanical properties and excellent physical structure to provide force support [59,60]. These biomaterials can also compensate for physical properties, such as mechanical strength, stiffness, viscoelasticity, and surface topography [61]. Commonly employed biomechanical scaffolds for cartilaginous joints encompass inorganic materials, ECM substrates, polymers, metals, and their respective composites. Given the comprehensive coverage in prior literature [24], we have summarized scaffold mechanical performance and novel techniques.

### 3.1 Mechanical properties

#### 3.1.1 Mechanical strength

**Fig. 4:**
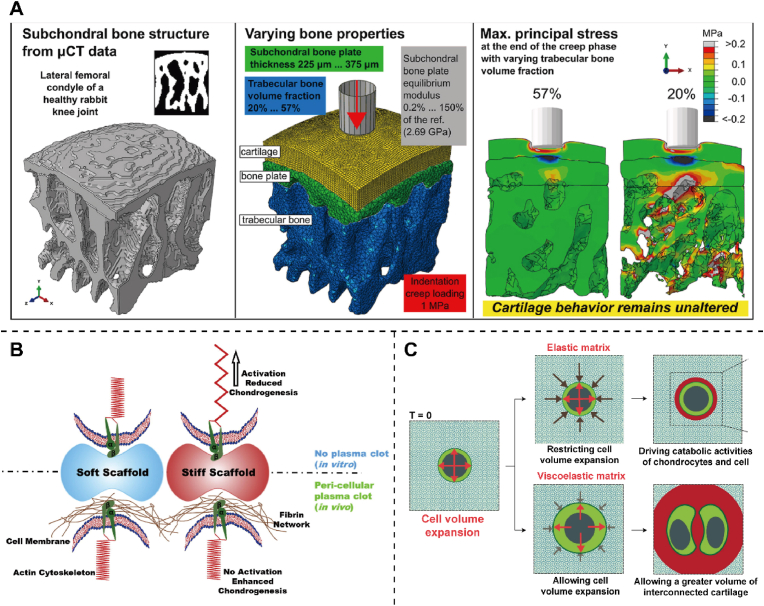
The mechanical support to the cartilage. (A) Effects of parameters such as trabecular bone volume fraction, subchondral bone plate thickness, and equilibrium modulus on osteochondral unit mechanics. Reproduced with permission [63]. Copyright 2022, Elsevier. (B) Schematic representation of the intricate interplay between stiffness and chondrogenesis, and how their responses differ in the presence and absence of plasma clots in vitro and in vivo. Reproduced with permission Reproduced with permission [67]. Copyright 2016, Elsevier. (C) The phenotype of chondrocytes is influenced by hydrogel stress relaxation, which occurs when the hydrogel resists volume change. This resistance ultimately limits the expansion of chondrocytes and the cartilage matrix formation. Reproduced with permission [71]. Copyright 2017, Springer Nature.

The injured cartilage can withstand less load and pressure and lose a lot of mechanical properties [62]. Changes in early injured subchondral bone structure minimally influence cartilage mechanics during creep indentation loading, with noticeable effects on cartilage mechanics only occurring in the presence of an unrealistically soft subchondral bone plate [63] (Fig. 4A). The simultaneous achievement of high stiffness, toughness, and rapid recovery poses a challenge, as these properties often conflict. Current technologies face difficulties in engineering scaffolds that are both stiff and tough to accurately replicate the mechanical attributes of cartilage-like stiff tissues [64]. Biomaterials should be high enough to withstand the load and stress of normal human activities and possess certain toughness and ductility. In arthroscopic cartilage repair applications, the ability of the hydrogel to solidify quickly underwater and bind firmly to the surrounding tissue is critical to withstand the pressure of arthroscopic irrigation (about 10 kpa). Hua et al. [65] reported a hybrid photocrosslinked (HPC) dual-network (DN) hydrogel manufactured by photoinduced radical polymerization and photoinduced imine crosslinking for autologous chondrocyte implantation (ACI). The mechanical properties of the nitrobenzyl group grafted hyaluronic acid into a double network hydrogel were greatly enhanced. The mechanical energy dissipation of the DN structure enhances the mechanical strength of HPC hydrogels, resulting in an increase in the hydrogel's ability to adhere to tissue, reaching up to approximately 2 MPa. Furthermore, the reinforced bonding between the HPC hydrogel and tissue allows for higher interfacial toughness by absorbing significant mechanical energy. Experiments on mice show that subcutaneous HPC hydrogel implantation containing chondrocytes can regenerate cartilage. Therefore, the rapid gelation, excellent mechanical properties and strong adhesion characteristics of HPC hydrogels are conducive to developing water-injection arthroscopic surgery. Furthermore, the mechanical properties of the composite may bring about changes in cell activity, providing an idea to change the physical properties for future therapeutic work.

#### 3.1.2 Stiffness

Matrix stiffness is a critical determinant of cellular differentiation. Specifically, prior research has demonstrated that soft scaffolds with a stiffness below 2–3 kPa can promote chondrogenesis and cellular aggregation. In contrast, stiffer scaffolds greater than 10 kPa can impair chondrogenesis in vitro [66]. In the present study, Arora et al. [67] investigated the effect of scaffold stiffness on in vitro chondrogenesis using gelatin scaffolds with soft (∼1.5 kPa) and stiff (∼40 kPa) properties under conditions of pericellular plasma clotting and non-clotting. Chondrocytes demonstrate efficient condensation and secretion of cartilaginous matrix when cultured on soft scaffolds. Compared to stiff scaffolds without pericellular plasma clotting, soft scaffolds and soft and stiff scaffolds with pericellular plasma clotting exhibit significantly higher levels of col II, chondroitin sulfate, and aggrecan deposition (Fig. 4B). Scaffold stiffness and other environmental cues are crucial in regulating chondrogenesis and cellular differentiation. Cells are susceptible to the hardness of their surroundings, and adjusting the hardness of materials can significantly impact cell growth, proliferation, and differentiation. The interaction between chondrocyte and hydrogel stiffness is still worth investigating. Zhou et al. [68] prepared hybrid hydrogels with adjustable stiffness based on ferrooxide magnetic nanoparticles (Fe_2_O_3_) and methacrylate gelatin (GelMA) through chemical crosslinking. Due to the synergy effect of hydrogel stiffness and trace iron ion releasing, the composite hydrogel can affect mitochondrial oxidative phosphorylation in chondrocytes [21]. This change was attributed to the generation of cellular lipid metabolites and breakdown of cellular lipids and may have activated PPARα signaling. Appropriate material stiffness is of utmost importance in cell culture and biomedical research. Chondrocytes exhibit adaptive responses to prolonged biomechanical stimuli. Material stiffness can impact chondrocyte differentiation and matrix synthesis. Joint cartilage damage and degeneration are commonly linked to stiffness. Excessive stiffness may induce improper stress distribution, hastening cartilage degradation and hindering normal joint biomechanics [69]. Engineers designing artificial joints must account for material stiffness to mimic natural joint biomechanics. Imbalances in artificial joint material rigidity may cause patient discomfort and premature joint failure. In conclusion, material stiffness significantly affects cartilage, influencing its biomechanical traits, cellular responses, and overall joint health [70].

#### 3.1.3 Viscoelasticity

Articular cartilage can withstand loads of up to 100 MPa, enduring millions of loading-unloading cycles with minimal fatigue and swiftly recovering its shape upon unloading. These unique mechanical characteristics are achieved through an intricate network of collagen fibers and proteoglycans within articular cartilage [64]. The viscoelasticity of materials plays a vital role in chondrocyte culture, providing better cell support and growth environment [72]. However, lower viscoelasticity can increase the ability of cell migration and diffusion, which is suitable for studying aspects such as cell migration and tissue regeneration [14]. Elastic chondrocyte hydrogels can be used as cartilage tissue equivalents to repair damaged cartilage. However, their elastic stress may alter the phenotype of chondrocytes and limit cartilage matrix formation. Lee et al. [71] studied cultured viscoelastic hydrogels for 3D chondrocytes with stress relaxation and creep over time. The rate of relaxation affects the volume changes of the interconnected cartilage matrix. In the slow relaxation hydrogel, the limitation of chondrocyte volume expansion due to elastic stress leads to an increase in the secretion of IL-1β, which up-regulates significantly genes related to cell death and cartilage degeneration [73] (Fig. 4C). Polymeric scaffolds are crucial in tissue engineering, providing a platform for cell growth and proliferation. Lin et al. [74] prepared PEGylated poly (glycerol sebacate) scaffolds with controllable crosslinking degrees and hierarchical macro-/micro-porosities. It exhibits remarkable potentials in promoting chondrogenic differentiation, maintaining chondrocyte phenotype, and enhancing cartilage matrix secretion. The viscoelasticity of the scaffold influences cellular processes including adhesion, migration, proliferation, and differentiation, significantly modulating the overall biomechanics of cartilage [75]. Excessive scaffold viscosity or elasticity can result in imbalanced stresses during movement, heightening the risk of cartilage degradation. Analogous to material stiffness, scaffold viscoelasticity may induce adaptive changes in cartilage under prolonged biomechanical stimuli, involving alterations in cell activity, matrix synthesis, and overall tissue structure.

#### 3.1.4 Surface topography

Cartilage serves as an effective lubricant for joints, minimizing direct bone-to-bone contact and reducing joint wear and tear [76]. However, cartilage injury results in losing the protective layer, leaving the joint surface vulnerable to damage and erosion. As a result, joint friction and wear increase, and bone-to-bone contact can occur, leading to further damage [77]. Thus, restoring cartilage's protective properties is crucial for effective cartilage repair.

**Fig. 5:**
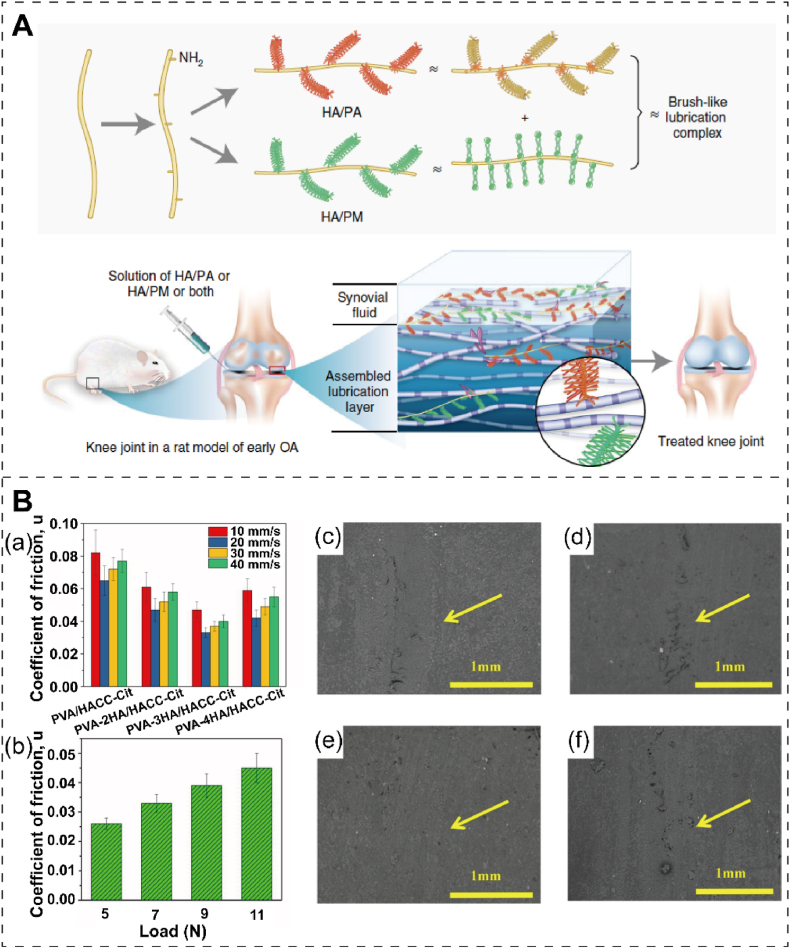
The improved lubrication and friction property to cartilage. (A) Schematic representation of brush nanofibers to enhance lubrication of damaged human cartilage. Reproduced with permission [78]. Copyright 2021, Springer Nature. (B) The average friction coefficients of various hydrogels were examined at different sliding speeds (a) and the PVA-3HA/HACC-Cit hydrogel under varying loads (b). Surface morphologies of PVA/HACC-Cit (c), PVA-2HA/HACC-Cit (d), PVA-3HA/HACC-Cit (e), and PVA-4HA/HACC-Cit (f), were observed after undergoing a 15-min friction test in 25 % fetal bovine serum with a sliding speed of 30 mm/s. Reproduced with permission [80]. Copyright 2020, Elsevier.

Effective tribological properties can mitigate wear of repair materials and cartilage tissue damage, highlighting the importance of lubricating properties. Furthermore, this lubrication effectively facilitates the movement and positioning of the repair materials within the cartilage [77]. Brush cartilage with lubricins and lipids combined with nanofibers with a HA skeleton plays a lubricating role in healthy cartilage. Xie et al. [78] combined sulfonate polymers and phosphocholine polymers with an acetyl HA skeleton to jointly enhance cartilage regeneration in early arthritis in a rat model. These biomimetic brush nanofibers can form a lubricating layer on the surface of cartilage, effectively reducing the friction coefficient to the low-level state of natural cartilage. Intra-articular injection of nanofibers into the articular cavity of rats with osteoarthritis abolished osteoarthritis and led to cartilage regeneration within 8 weeks (Fig. 5A). The surface nano-roughness can facilitate cell adhesion. Ding et al. [79] developed lacunar hyaluronic acid microcarriers (LHAMC) under mechanotransductive conditions, which enable the production of stable hyaluronic acid (HA) *N*-hydroxy succinimide ester (NHS-ester) that facilitates the regeneration of hyaline cartilage. The microcarriers are formed through the linkage of carboxyl-functionalized HA to collagen type I via amide-crosslinking, and their concave surface is produced through gas foaming via ammonium bicarbonate. When chondrocytes are cultured on LHAMC in a 3D environment, they can remodel the extracellular matrix, resulting in hyaline cartilaginous microtissue regeneration while preventing chondrocyte dedifferentiation.

The frictional properties of materials have a significant impact on cartilage repair. During cartilage repair, the friction between repair materials and surrounding cartilage tissues can affect fixation, stability, and functional recovery [81]. Gan et al. [80] developed physical crosslinked double-network hydrogel with an organic and inorganic composite, polyvinyl alcohol-nano hydroxyapatite/2-hydroxypropyltrimethyl ammonium chloride chitosan (PVA-HA/HACC-Cit). Due to its unique dual physical crosslinked structure, the PVA-HA/HACC-Cit has excellent high fracture tensile stress of 2.70 MPa and 14.09 MJ/m3 toughness. The inclusion of nanometer-scale hydroxyapatite endows DN hydrogel with a low friction coefficient, exceptional wear resistance, and cytocompatibility (Fig. 5B). In joint mechanics, surface friction plays a pivotal role in facilitating normal joint movement. Optimal friction levels are essential for preserving the regular motion and stability of joints. Conversely, immoderate or uneven friction has the potential to induce surface wear and damage to cartilage, thereby exacerbating joint pathologies. When selecting and designing cartilage repair materials, the friction force between the balanced material and the surrounding cartilage tissue needs to be considered to achieve optimal cartilage repair outcomes and functional recovery.

### 3.2 Stimuli-responsive smart scaffolds

Articular cartilage is inherently insensitive to self-repair in its native environment [[82], [83], [84]]. When employing traditional surgical approaches to treat joint cartilage injuries, the substantial incisions pose challenges for effective recovery. However, biomaterials can alter the surrounding physical signals, harnessing electrical, thermal, optical, magnetic, enzyme, and other stimuli to stimulate cartilage repair activities (Table 1).

**Table 1:** Stimuli-responsive smart biomaterials for the types of response, materials, principles, and characteristics.

Type of response	Material	Principle	Characteristic	Ref.
Electricity	CA–CS–HA and PCL/PDMS/PCL–FA	Converting mechanical energy into electrical energy to promote chondrocyte proliferation	Implantable; self-powered, anti-interference; highly sensitive; intelligentically degradable	[87]
	Nanofibrous scaffold of PLLA	Generating adjustable piezoelectric charges under applied force; altering calcium signaling pathways to induce endogenous TGF-β	Biodegradable; controlled mechanical activation	[89]
Temperature	PAF-PEG-PAF	The enlarged pore size and enhanced mechanical strength generate the cartilage and reduce the formation of fibrous tissue	Excellent temperature responsiveness; minimally invasive implantation in situ	[94]
	stereocomplex 4-arm PEG–PLA	The cartilaginous-specific matrix, biomechanical property, and gene expressions	Improved mechanical properties; larger pore size; better chondrocyte adhesion	[29]
NIR light	E@Au–Ag	Anti-oxidative stress to reduce chondrocyte apoptosis; up-regulating col II expression in chondrocytes and decreasing apoptosis marker proteins *p*-caspase-3 and MMP13 expression	Antibacterial; NIR-sensitive; synergistic therapy	[95]
Light	CM-NTU	Increasing intracellular ATP and NADPH levels and improving anabolism in degenerating chondrocytes	The natural photosynthetic system controls cell behavior; the cross-species system	[100]
Magnetism	WY-CMC-MnO_x_	Promoting chondrogenesis of MSCs	Precise ability to target cartilage; ultrasmall sizes	[96]
	MNPs (CAG)	Upregulating the chondrogenesis-related genes of COL2A1 and ACAN	3D magnetic scaffolds; operating under a dynamic magnetic field	[101]
Enzyme	PEG-KGN	Quickly fill with bone marrow blood shortly after implantation, enabling maximal absorption and retention of ESPCs	Fast absorption of protein solution through capillarity	

#### 3.2.1 Electricity

**Fig. 6:**
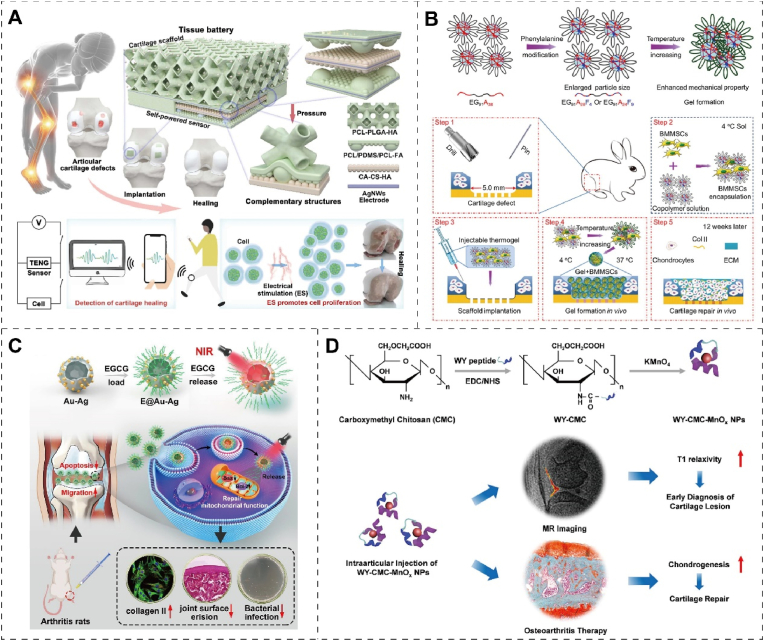
The biomaterials respond to physical signals for cartilage treatment. (A) Schematic representation of tissue batteries formed by combining TENG sensors and porous 3D smart scaffolds for cartilage therapy. Reproduced with permission [87]. Copyright 2023, Elsevier. (B) Schematic representation of the synthesis of triblock copolymer thermal polypeptide hydrogels with different proportions of alanine and phenylalanine to promote cartilage regeneration. Reproduced with permission [94]. Copyright 2018, Elsevier. (C) Schematic diagram of near-infrared light-sensitive composite nanomaterials for cartilage protection and regeneration. Reproduced with permission [95]. Copyright 2022, Elsevier. (D) Schematic illustration of the synthesis of WY-CMC-MnO_x_ nanoparticles, and their application in magnetic resonance (MR) imaging and cartilage repair. Reproduced with permission [96]. Copyright 2022, Elsevier.

Low-intensity electrical stimulation can promote cell metabolism, increase the synthesis of ATP, and thus promote cell proliferation. Electrosensitive biomaterials can respond to external electric signals and convert electrical energy [85]. Excellent tissue wound treatment can be achieved by using the electrical activity characteristics of the organism [86]. Yue et al. [87] combined a triboelectric nanogenerator (TENG) sensor with a porous 3D smart scaffold to form a tissue battery. Collagen aggregate-chitosan-hydroxyapatite (CA–CS–HA) and poly (ε-caprolactone) -polydimethylsiloxane-poly (ε-caprolactone) -fluorapatite (PCL/PDMS/PCL–FA) served as positive and negative electrodes, respectively, forming a sealed negative/positive/negative sandwich structure. The TENG sensor exhibits a remarkable sensitivity of 52.5 V Mpa^−1^ within the 0–1.8 MPa, enabling the tissue cell to monitor cartilage repair status in real-time and situ. In vitro and in vivo experiments show that the tissue battery can promote the proliferation of chondrocytes by converting mechanical energy into electrical energy, thereby treating cartilage defects and shortening the repair period (Fig. 6A). Electrical stimulation of materials for tissue repair is a promising method for bioelectronic implants, which will have a broad prospect in intelligent medicine. Electrical stimulation can increase the calcium ion channel opening of the cell membrane, thereby increasing the concentration of intracellular calcium ions, which in turn affects cell metabolism and motility [88]. In addition, electrical stimulation can also cause vibration and movement of cells by affecting their charge distribution. Liu et al. [89] designed a biodegradable nanofibrous scaffold of poly (*l*-lactic acid) (PLLA) that can produce electrical stimulation to promote chondrogenesis and regeneration under the application of force or joint loading. The PLLA scaffolds can generate piezoelectric charges that can be adjusted according to the applied force or joint load. Furthermore, they can modify calcium signaling pathways, leading to endogenous TGF-β induction. The scaffolds also facilitate the recruitment and migration of cells, promote the adsorption of extracellular proteins, and enhance chondrogenesis both in vitro and in vivo. In a rabbit cartilage defect model, articular cartilage treated with piezoelectric scaffolds showed hyaline cartilage regeneration, more col II and chondrocytes. Therefore, Electrical stimulation can affect cell metabolism and motility by affecting ion channels of the cell membrane.

#### 3.2.2 Temperature

Traditional hyperthermia methods such as hot packs, faradic baths, and infrared saunas have long been recognized for their palliative effects in relieving inflammation pain [90]. However, research has shown limited efficacy of these traditional heat treatments in managing cartilage functions and structures. On the contrary, an auspicious direction in tumor treatment lies in localized hyperthermia, facilitated by nanoparticle-mediated techniques like magnetocaloric or photothermal therapy [91]. This refined medical approach has exhibited synergistic outcomes when integrated with chemotherapy, leading to the inception of a pioneering tumor therapy termed thermochemotherapy [92]. Gang et al. [93] engineered a multifunctional hydrogel (DN-Fe-MTX-TGFβ1) by incorporating a first-line antirheumatic drug (methotrexate; MTX) and TGF-β1 into a nano-Fe_3_O_4_ composite chitosan-polyolefin matrix. The hydrogel's long-term release capability and magnetocaloric properties substantiate its potential in delivering sustained local thermochemotherapy. Local drug-magnetocaloric combination therapy effectively mitigates RA-induced decrease of Col II and cartilage degradation, thus promoting the repair of RA-induced cartilage defects. Magnetothermal therapy increased the proportion of healing subtype M2b macrophages, enhancing the anti-inflammatory capacity of MTX-loaded hydrogels (Fig. 6B).

#### 3.2.3 Light

Biological materials transform light energy to heat energy, on the other hand can also act as a non-intrusive way through biological material indirect role in energy metabolism of cartilage [97]. Photosensitive biomaterials could achieve cartilage repair through photothermal and photochemical effects under light stimulation [98]. Near-infrared (NIR) fluorescence is non-invasive, allowing controlled release in vitro. NIR-sensitive nanoenzyme composites can effectively protect cartilage [99]. The NIR-sensitive Epigallocatechin gallate (EGCG) can be used as an enzyme-sensitive active nanomaterial modified in Au–Ag nanocapsules (E@Au–Ag). E@Au–Ag can have an excellent photothermal effect under NIR irradiation, raising the intra-articular temperature to 46.6 °C and promoting the release of EGCG. Molecular biology experiments indicate that E@Au–Ag up-regulated the expression of col II in chondrocytes, while decreasing the expression of apoptosis marker proteins *p*-caspase-3 and MMP13. These changes are conducive to facilitating cartilage migration and regeneration. Furthermore, this multifunctional phototherapy nanoplatform with antibacterial properties can effectively prevent infection [95] (Fig. 6C). The limitation of intracellular anabolism is one of the reasons for the pathological process in vivo. Under pathological conditions, ATP and NADPH concentration levels are unbalanced, impairing intracellular anabolism. Chen et al. [100] developed a photosynthetic system derived from plants that utilizes nanothylakoid units (NTUs) technology. This cross-species application system is to encapsulate specific mature chondrocyte membranes (CM). The light irradiation of CM-NTU increases intracellular ATP and NADPH levels, improving anabolism in degenerating chondrocytes. This systematic correction of energy imbalance can potentially improve cartilage homeostasis and prevent the pathological progression of cartilage diseases. Natural photosynthetic systems can control cellular anabolism by independently providing metabolic carriers and critical energy. It enlightens the promise of composite biomaterials and biological organisms to treat degenerative diseases by modulating the anabolic aspects of cells.

#### 3.2.4 Magnetism

Magnetic-sensitive biomaterials can sense, respond, and utilize the surrounding magnetic signals to achieve physiological functions such as localization, navigation, probing, and communication [[102], [103], [104]]. However, neither clinical drugs nor MR contrast agents can detect and repair simultaneously. Lin et al. [96] synthesized carboxymethyl chitosan (CMC) assisted manganese oxide (MnO_x_) nanoparticles by binding CMC to a chondroscope-targeting peptide (WYRGRL, called WY). Due to its ultra-small size and precise ability to target cartilage, WY-CMC-MnO_x_ significantly improves the quality of MR imaging of cartilage lesions. In contrast, gadolinium-diethylenetriamine pentaacetic acid, a clinically used assay, failed to detect cartilage lesions. In addition, WY-CMC-MnO_x_ promotes chondrogenesis of MSCs, thereby treating OA through effectively enhanced cartilage regeneration following injection of the medial articular meniscus in a rat model (Fig. 6D). The fabrication of 3D magnetic scaffolds represents a novel approach to enhancing MSCs differentiation for osteochondral repair. Zhang et al. [101] incorporated magnetic nanoparticles (MNPs) into electrospun gelatin nanofibers to produce 3D magnetic scaffolds. Mechanical stimulation of the embedded MSCs was induced by positioning the scaffolds near a rotating magnet with a frequency of 0.5 Hz, resulting in spatial confinement and fluid flow. Notably, the magnetic scaffolds with citric acid-coated MNPs selectively up-regulated chondrogenesis-related genes (COL2A1 and ACAN), indicating their potential for enhancing chondrogenesis. Before in vivo implantation, the MSCs were chondrogenically preconditioned within the scaffolds under a dynamic magnetic field, resulting in superior osteochondral repair. The above biomaterials vary in physical signal-sensing mechanisms, but they all provide important ideas for cartilage repair, enlightening researchers to design novel and sensitive materials. enzyme.

#### 3.2.5 Enzyme

Enzyme-responsive release systems are practical smart drug delivery systems designed based on the specific properties of enzymes, such as their high expression levels in conditions like inflammation, cancer, and infection. In the context of in-situ cartilage tissue engineering, Kartogenin (KGN) dosages can be adjusted using enzyme-responsive systems for cell recruitment and differentiation. Yu et al. [105] developed an in-situ prepared porous cartilage tissue engineering scaffold with enzyme-responsive KGN release, facilitating the recruitment of endogenous stem/progenitor cells (ESPCs) through chemical cues. An enzyme-responsive polymeric scaffold was fabricated by modifying poly (lactic-*co*-glycolic acid) (PLGA) with PEG-KGN and PCL. This scaffold immediately absorbs and retains ESPCs from the bone marrow upon implantation and promotes their migration to the defect site. MMP-2 was found to accelerate PEG-KGN release. Chondrogenic differentiation was achieved by releasing KGN based on the local inflammatory microenvironment, with high or low doses of PEG-KGN sequentially released according to the severity of inflammation. Chondrocytes can either enhance cartilage synthesis metabolism by producing tissue components or drive breakdown metabolism by secreting aggrecanases and collagenases, with MMP-13 being one of the therapeutic targets. Downregulating MMP-13 via small molecule inhibitors or inhibiting its enzymatic activity has shown promise in preventing or reversing OA cartilage degeneration. In the early stages of OA development, MMP-13 overexpression leads to extracellular matrix degradation, contributing to cartilage degeneration. Liang et al. [106] engineered cartilage cell-targeted exosomes (CAP-Exo) by fusing the *N*-terminal of the Lamp2b protein on exosome surfaces with a cartilage cell-affinity peptide (CAP). Hybrid CAP-Exo penetrated deep into the cartilage matrix of arthritic rats, delivering Cas9 sgMMP-13 plasmids to chondrocytes, effectively downregulating MMP-13 expression within chondrocytes, and reducing the hydrolytic degradation of extracellular matrix proteins in cartilage.

### 3.3 3D-printed scaffolds

Rapid advancements in engineering and biotechnology have ushered in new possibilities for cartilage regeneration. Using 3D biopanning, it is now feasible to create customized scaffolds that possess a meticulously controlled structure, anisotropy, and cell distribution closely resembling that of native cartilage tissue. 3D bioprinting is a technology that uses natural or synthetic materials to fabricate scaffold structures, imitating the structure and function of natural tissues [[107], [108], [109]]. 3D printing technology provides early mechanical support to the growing tissues by enabling the production of multi-layered biodegradable biological patches. The material gradually degrades over a period of time, allowing chondrocytes to secrete extracellular matrix, which eventually fills the scaffold. The secreted extracellular matrix provides mechanical stability after the degradation of the biomaterial. An ideal biomaterial should be non-toxic, with degradation products that are controllable and absorbable [110]. Meanwhile, the rheological properties of the biomaterial are also significant for ensuring good mechanical stability and high cell survival rate [111]. Specifically, selecting biological ink for 3D printing is crucial for creating complex, functional tissues that can be used for various applications [112]. 3D printing cartilage may become a viable option for treating many cartilage-related conditions, such as osteoarthritis and traumatic injuries.

**Fig. 7:**
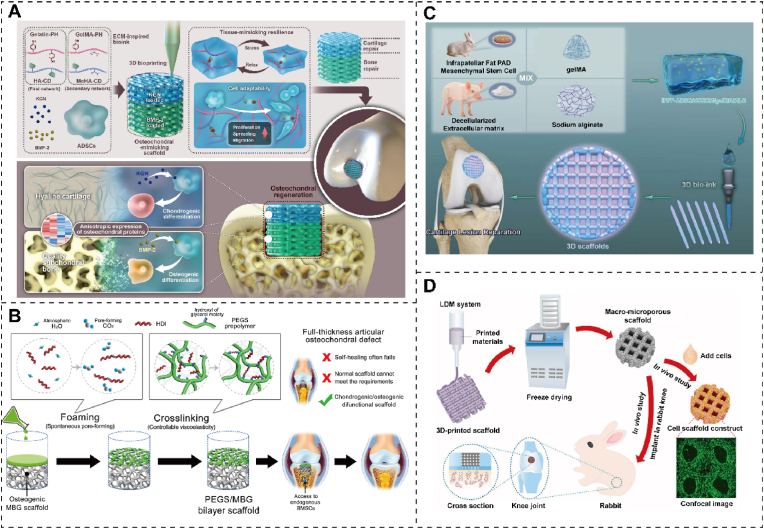
Replacement effect of 3D printed cartilage. (A) Schematic depicting enhanced osteochondral defect healing, marked by hyaline cartilage and high-quality subchondral bone formation, achieved through an ECM-inspired natural scaffold. Reproduced with permission [116]. Copyright 2023, Elsevier. (B) Schematic depicting the fabrication process of the chondrogenic/osteogenic PEGS/MBG bilayer scaffold, and its utilization in the context of a full-thickness articular osteochondral defect. Reproduced with permission [74]. Copyright 2020, Elsevier. (C) Schematic illustration of 3D bioprinted scaffolds and cartilage repair. DCECM, IPFP-ADSC, GelMA, and alginate (ALG) co-exist with 3D bio-ink for articular cartilage repair. Reproduced with permission [117]. Copyright 2022, Elsevier. (D) Schematic preparation of the WPU-ECM hybrid scaffold and cartilage repair in cells and rabbit models. Reproduced with permission [118]. Copyright 2021, Elsevier.

Natural materials, serving as biocompatible bioinks, offer excellent compatibility and can effectively substitute for cartilage. Collagen, a primary component of cartilage's ECM, contains abundant arginine-glycine-aspartic acid sequences and MMP cleavage sites, facilitating cell adhesion and remodeling. Dai et al. [28] strategically modified natural biopolymers to create a reversible host and a rigid covalent network, resulting in a novel ECM-mimicking scaffold composed of natural polysaccharides and peptides. Additionally, it precisely incorporates cartilage and bone-forming agents into specific scaffold regions, accelerating high-quality cartilage-to-bone regeneration in rabbit bone cartilage defects (Fig. 7A). The ECM scaffold exhibits a robust capacity to promote transparent cartilage formation and features a typical porous structure, sufficient mechanical strength, good elasticity, and cartilage-specific ECM deposition.

Due to its 3D structure and resemblance to natural bone matrix, particularly in osteoinductive and biomechanical properties, decellularized bone has emerged as an ideal scaffold for bone engineering. Incorporating type I collagen into a collagen-based bioink has demonstrated increased cell viability and improved stability of tissue architecture, resulting in a higher ECM content in the generated tissues. This approach shows great potential for creating functional tissues across various biomedical applications [113]. The graded macroporous structure presents an innovative therapeutic avenue for addressing cartilage defects and holds promise for application in other tissue defect scenarios. In the realm of cartilage tissue regeneration, decellularized cartilage ECM (DCECM) emerges as a prime biological scaffold, capable of serving as a versatile carrier for different bioactive components, such as col II, chondroitin sulfate, as well as specific growth factors and cytokines, guiding the formation of cartilage tissue. 3D tissue engineering scaffolds play a pivotal role in providing the necessary support framework and mechanical strength, crucial for the proliferation and maturation of seeded cartilage cells and the effective secretion of ECM components in vivo.

In contrast to their natural counterparts, synthetic scaffolds offer precise control over their physical properties, such as viscoelasticity, hardness, and stiffness. Low cross-linking viscoelastic polyethylene glycol sebacate (PEGS) has been demonstrated to significantly stimulate chondrogenic differentiation, maintaining chondrocyte phenotypes and enhancing cartilage matrix secretion, as compared to highly cross-linked elastomeric polymers. Lin et al. [74] introduced a solvent-free polyurethane cross-linking method that spontaneously forms pores at room temperature, yielding PEGS scaffolds with controllable cross-linking degrees and graded macro/microporosity. Building upon this, a dual-function PEGS/MBG bilayer scaffold, combining low cross-linking viscoelastic PEGS with osteoinductive mesoporous bioactive glass (MBG), was successfully used to reconstruct well-integrated articular hyaline cartilage and its underlying bone in a full-thickness osteochondral defect model in vivo (Fig. 7B). Synthetic scaffolds can finely tune their biodegradability, ensuring the sustained release of bioactive factors. Cells can sense their dynamic chemical and physical microenvironments. They translate material degradation or remodeling-induced dynamic stimuli into biochemical and biological responses, ultimately achieving the desired cellular functions and tissue maturation. A comprehensive investigation of the impact of stepwise dynamic hydrostatic pressure (DHP) on in vitro engineered cartilage and its subsequent in vivo regeneration in cartilage defects was conducted for the first time using a composite scaffold of regenerated silk fibroin protein and bacterial cellulose (BC) nanofiber ribbons. Compared to conventional static culture, DHP positively enhanced chondrocyte growth and cartilage-specific matrix deposition [114]. Pei et al. [115] devised a 3D-printed poly (ε-caprolactone) (PCL) scaffold modified with PLGA nanoparticles for the gradual release of insulin, facilitating osteogenic differentiation of rabbit bone marrow-derived mesenchymal stem cells and proliferation of chondrocytes.

The availability of biocompatible synthetic materials for 3D printing remains somewhat limited. Commonly employed printable materials such as polylactic acid (PLA), PLGA, and PCL often necessitate elevated printing temperatures or organic solvents. High-temperature processing can compromise material integrity, leading to reduced mechanical performance. Additionally, concerns over residual solvents in the final product may hinder broader biomedical applications. Wu et al. [117] formulated bioinks containing infrapatellar fat pad-derived ADSCs (IPFP-ADSCs), resulting in a 3D bioprinting scaffold (IPFP-ADSC/DCECM/GelMA/ALG). This 3D DCECM scaffold implantation with IPFP-ADSCs provided an advantageous cell matrix, promoting chondrogenesis and differentiation, ultimately enhancing cartilage repair in a rabbit cartilage defect model (Fig. 7C). This biocompatible 3D scaffold capitalizes on the strengths of DCECM and IPFP-ADSCs, demonstrating remarkable in vitro cartilage-forming capacity and in vivo cartilage repair potential. Chen et al. [118] harnessed a low-temperature deposition and phase separation approach for 3D printing, yielding a hybrid scaffold composed of water-based polyurethane (WPU) and acellular cartilage ECM featuring a stratified macroporous structure. The inclusion of ECM optimizes WPU properties, including hydrophilicity, porosity, and bioactive elements. Consequently, WPU-ECM scaffolds exhibit superior cell proliferation, adhesion, and differentiation capabilities compared to single-component scaffolds. In a rabbit model, 6 months post-implantation, the repaired cartilage within WPU-ECM scaffolds demonstrated equivalent biomechanical properties and histomorphology to normal cartilage (Fig. 7D). Melt electro-writing technology offers precise control over scaffold design and porosity [40]. Han et al. [116] devised a technique that enhances printability and formability by blending gelatin with polylactic acid-glycolic acid to create high-concentration, high-viscosity printing inks. They employed inkjet technology to fabricate a composite scaffold loaded with hydroxyapatite and TGF-β1, which, when implanted at a cartilage injury site following microfracture surgery to generate high-quality BMSCs, effectively repaired the damaged cartilage. Yang et al. [119] utilized 3D co-printing technology to construct a tissue engineering scaffold conducive to cartilage release and repair. Co-printing GelMA and PCL with extracellular matrix components ensured mechanical robustness. In a clinically relevant sheep femoral condyle articular cartilage defect model, incorporating TGF-β3 microspheres into the mimic cartilage scaffold guided the differentiation and migration of endogenous stem cells, thereby enhancing cartilage tissue repair. Introducing cytokines into bioink necessitates stringent control over concentration and stability to mitigate adverse effects on cells.

Reconstructing complex tissues using bioceramics combined with bioink bioprinting is a promising strategy to stimulate cell differentiation in multiple tissues [120]. Their chemical composition and crystalline structure are similar to human tissues, reducing the risk of immune reactions and rejection. The bioceramics surfaces exhibit biological activity, which can facilitate cell adhesion and growth, and accelerate the repair and regeneration of bone tissue [121]. Furthermore, bioceramics can be fabricated to possess pore structures and morphologies resembling natural bone tissues, which favor bone cell colonization and growth of bone cells [122]. The anisotropic tissue lineage and physiological properties of osteochondral tissue remain challenges for cartilage regeneration. Qin et al. [123] developed a system of bilayer co-culture scaffolds formed by 3D bioprinting to simulate osteochondral tissue to replace osteochondral for regeneration. The top of the co-culture scaffold was a hydrogel bioink loaded with chondrocytes, and the bottom was a lithium Mg–Si bioceramics bioink loaded with MSCs. Bioinks containing Li–Mg–Si biocrystals induce specific differentiation of various cells by releasing bioactive ions, which can mimic bone and stimulate cartilage and subchondral bone regeneration. 3D bioprinted co-culture scaffolds have demonstrated their potential for regenerating osteochondral tissue by stimulating chondrogenic differentiation in vitro and effectively repairing osteochondral defects in vivo. However, further research is needed to refine the techniques and materials used in 3D printing cartilage, as well as to address regulatory and ethical considerations associated with using 3D printing in medicine.

### 3.4 Cartilage bionic scaffolds

Cartilage repair scaffolds are a critical component of regenerative medicine, providing a specific spatial structure and bearing seed cells for tissue engineering. These scaffolds are essential for replacing the function of damaged tissues and organs [124]. Cartilage-related scaffolds provide the geometry and mechanical properties required for chondrocyte growth, potentially affecting cell differentiation and cell surface factor receptor expression [125]. An ideal scaffold should have excellent biocompatibility, a highly porous structure, biodegradability, plasticity, and mechanical properties. Therefore, meticulous selection and preparation of cartilage scaffold materials are significant for repairing cartilage injuries.

#### 3.4.1 Porosity

**Fig. 8:**
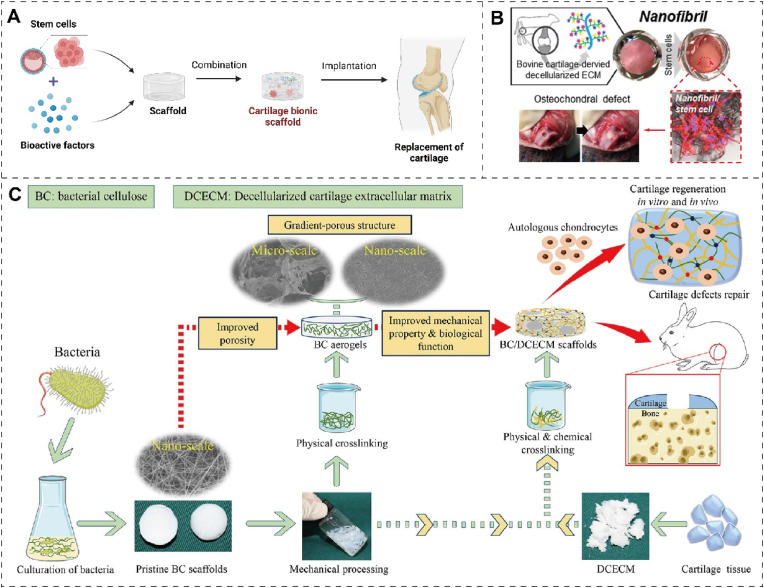
Tissue engineering scaffolds as biomimetic structure for cartilage repair. (A) Schematic Diagram of construction of bionic cartilage scaffold with the function of replacing cartilage. The figure was created with https://app.biorender.com/. (B) Schematic representation of DECM composite scaffolds loaded with ADSCs nanofibers filling cartilage defects for cartilage regeneration. Reproduced with permission [130]. Copyright 2021, Elsevier. (C) Schematic diagrams of the procedure of 3D hierarchical porous scaffolds for cartilage tissue engineering, involving the design of scaffolds with biomimetic features that closely mimic the structure and biochemical environment of native cartilage. The scaffolds can be used as platforms for cartilage-related injuries and disorders. Reproduced with permission [128]. Copyright 2022, Elsevier.

An ideal scaffold should feature a nanofibrous morphology to facilitate cell adhesion, a porous structure to enable cell migration, and a surface topography that can influence cell scaffold and cell-cell communication [124,126]. Furthermore, it should exhibit biological functions to guide cell behavior and facilitate tissue regeneration. To address the lack of biological function of 3D nanoscale pore structures [127], Li et al. [128] developed a composite 3D hierarchical porous scaffold that imitates the structure and biochemical microenvironment for cartilage regeneration. Using freeze-drying techniques, they produced 3D-graded porous scaffolds using a combination of BC and DCECM). The composite scaffolds of BC/DCECM exhibit excellent mechanical properties due to the macroporous structure generated by physicochemical crosslinking. In environments with high humidity, the material's strong water absorption confers high resilience and memory retention. The polymeric scaffold demonstrated a marked increase in chondrocyte adhesion, proliferation, and extracellular matrix synthesis. In vitro and in vivo experiments demonstrated that the BC/DCECM scaffold achieved significant regeneration of new cartilage tissue with an external native cartilaginous appearance and histological cartilage-specific cavity formation (Fig. 8A). This study provides a promising and practical approach for clinically translating biochemically and structurally simulated scaffolds for cartilage regeneration. The drug can be dispersed in the polymer material to form microspheres. Lv et al. [129] prepared microspheres using hydrogel micropores and cobalt ion crosslinking, resulting in a GelMA sodium alginate double-network hydrogel with fine-structure micropores. Conjugation of microwells to injectable artificial cartilage grafts has been shown to up-regulate proteins and genes related to cartilage differentiation in vitro. In vivo hydrogel injection into cartilage defects and removal of some ions, the covalent crosslinking formation can form a stable gel and repair irregularly shaped cartilage defects. This study broadens the application of micropores and advances tissue engineering strategies by designing intelligent materials.

#### 3.4.2 Surface modification

Surface treatments can enhance cell adhesion and growth on scaffolds by introducing functional groups, bioactive molecules, and micro/nanostructures [131]. Jin et al. [130] prepared a cartilaginous decellularized extracellular matrix (DECM) coated with polymeric nanofibers as a scaffold material for cartilage repair. Polymeric nanofibers were modified superficially with poly (glycidyl methacrylate) (PGMA@NF), where the epoxy group of PGMA@NF can react with DECM. Thus, DECM-decorated nanofibers biochemically and structurally mimic the cartilage-specific microenvironment. The DECM-modified nanofibers, self-assembled with ADSCs through cell-directed binding, significantly up-regulated the expression of chondrogenic-related genes. The nanofiber composite scaffold loaded with ADSCs filled the cartilage defect, and the newly formed soft bone was compact and could achieve long-term regeneration for 12 weeks (Fig. 8B). Compared with the single material, the composite scaffold loaded with ADSCs after PGMA@NF treatment reconstructed the ECM matrix of cartilage and subchondral bone. Modifying the surface to hydrophilic, such as by introducing hydroxyl or amine groups, can promote chondrocyte adhesion and proliferation [132].

#### 3.4.3 Decellularization

Acellular scaffolds possess natural biocompatibility and bioactivity as an ideal scaffold material for cartilage tissue engineering [133]. The decellularized scaffolds reduce the immune reaction with the host immune system and improve graft survival. ACI or microfracture often result in an undesired mixture of hyaline cartilage and inferior fibrocartilage. Nie et al. [134] designed a decellularized living hyaline cartilage graft (DLHCG) based on acellular technology and cartilage tissue engineering. DLHCG maximizes the clearance of cellular components and promotes host-graft integration in situ. Living hyaline cartilage graft in vivo as a parallel control highlighted the effect of decellularization [135]. After 6 months, the pig knee implantation experiment demonstrated that the regenerated hyaline cartilage had excellent fine morphology, phenotype, composition, microstructure, and mechanical properties (Fig. 8C). The articular cartilage of DLHCG after transplantation has excellent compatibility and consistency with the natural cartilage of the host. Clinical trials of allogeneic DLHCG have also been demonstrated in large animal models of articular cartilage defects. Li et al. [136] developed a GelMA/HAMA hydrogel material with tunable mechanical properties and acellular cartilage matrix composite biomaterial successfully constructed by violet crosslinking. Composite materials can better mimic natural cartilage and have characteristics similar to cartilage tissue, such as high elasticity and water content. The ECM can also interact with chondrocytes, stimulating specific biological responses such as cell differentiation and tissue regeneration [137]. Zeng et al. [138] developed an excellent biocompatible injectable porcine cartilage-derived DECM hydrogel for repairing cartilage defects. DECM hydrogel can enhance the chondrogenic differentiation of human urine-derived stem cells and promote the secretion of the extracellular matrix without adding any chondrogenic-inducing factors.

## 4 Biologically active substances

Cartilage tissue comprises chondrocytes embedded in the ECM, primarily consisting of collagen and proteoglycans [139]. The avascular cartilage receives oxygen and nutrients through the surrounding tissues and joint fluid [140]. Unlike skeletal tissues, cartilage cannot repair itself, possessing the slow proliferation and regeneration rate of chondrocytes [103]. Exogenous intervention is required to accelerate the cartilage bioremediation process. Biomaterials derived from natural or synthetic tissues have excellent biocompatibility and do not cause rejection reactions [141]. Interventions applied outside the body, such as implanting bioactive factors, stem cells, extracellular vesicles, and organoids, can promote regeneration and repair damaged cartilage [142]. Thus, it is of great clinical importance to utilize cartilage bioremediation technology, which can ameliorate patient discomfort and reduce the occurrence of postoperative complications through a minimally invasive approach. Additionally, this technology can be tailored to individual patients based on the extent of cartilage damage, demonstrating strong plasticity and adaptability.

### 4.1 Bioactive factors

**Fig. 9:**
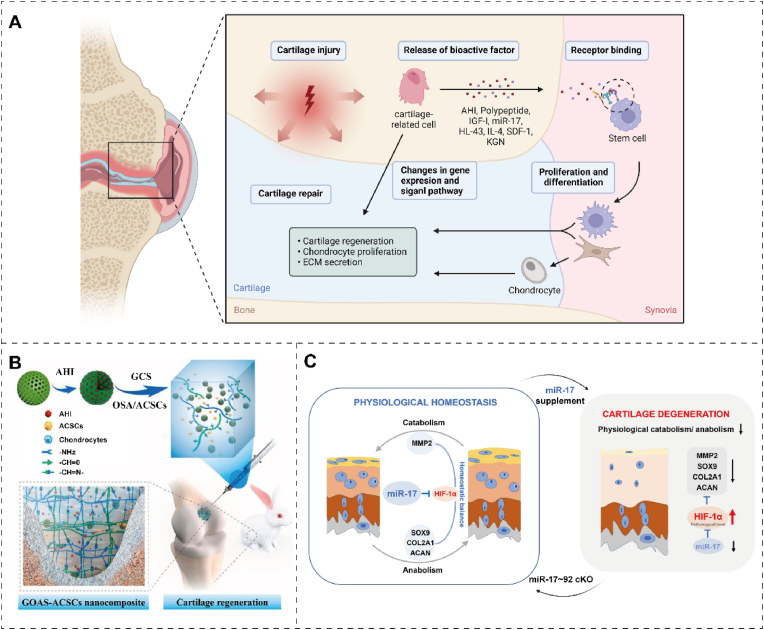
The role of bioactive factors in cartilage repair. (A) Schematic diagram of bioactive factor's role in repairing injured cartilage. The figure was created with https://app.biorender.com/. (B) Schematic illustration of cartilage regeneration through sustained AHI release by an injectable composite hydrogel loaded with AHI and mSiO_2_ NPs containing ACSCs. Reproduced with permission [157]. Copyright 2023, Elsevier. (C) Diagram of exogenous miR-17 or endogenous miR-17 induced by GDF-5 restoring cartilage homeostasis and preventing OA. Reproduced with permission [158]. Copyright 2022, Elsevier Springer Nature.

Bioactive factors are small proteins with biological activity that can promote proliferation, differentiation, matrix synthesis, cell migration, and anti-inflammatory effects by binding to specific receptors [143]. These mechanisms may be interactive, and different bioactive factors may utilize different mechanisms to promote tissue repair. Bioactive factors can be categorized as cytokines, chemokines, polypeptides, growth factors, and hormones [144]. Thus, when combined with specific biological materials, bioactive factors can promote cartilage tissue repair after trauma. (Table 2 and Fig. 9A).

**Table 2:** Bioactive factors in therapeutic target, principle, and characteristic.

Type of bioactive factors	Target of action	Principle	Characteristic	Ref.
AHI	ACSCs; ECM	Promoting ACSCs proliferation and differentiation and ECM production.	Excellent sustained release effect; injection in situ.	[157]
IGF-I	IL-1β; TNF-α	Decreasing expression of detrimental proinflammatory mediators (IL-1β, TNF-α)	Sequential releasing in a spatiotemporal manner.	[73,161]
Nap-FFG-GYGSSSRRAPQT	ADSCs	Activating IGF-1 receptor and up-regulating the expression of cartilage-related genes in ADSCs.	Substitution of self-assembled polypeptide polymers.	[152]
miR-17	MMP3/13; ADAMTS5; NOS2	Decreasing HIF-1α signaling to maintain physiological anabolic and catabolic balance.	Maintaining cartilage homeostasis and preventing OA simultaneously.	[158,162]
HL-43	EP4	Enhancing cartilage anabolism through Sox9 signaling and inhibiting it via STAT3 signaling.	Low toxicity; desirable bioavailability	[163,164]
IL-4	M1/M2 macrophage	Immune regulation on decellularized cartilage ECM scaffolds.	Precise and active immunomodulatory.	[146]
SDF-1 and KGN	CXCR4 receptors; runt-related transcription factor (RUNX)	Stem cell homing; stimulating RUNX1 expression.	Cell-free system; long-time sustained release.	[147]
KGN	PB-MSCs	Activating multiple cartilage-related genes and signaling pathways; promoting chondrogenic differentiation of PB-MSCs.	In situ gelation; sustained release.	[155]
SDF-1α and TGF-β3	MSCs; ECM	Increasing cell migration and matrix formation	Cell-free scaffold; dual-factor release	[153]

#### 4.1.1 Promotion of chondrocyte proliferation

Growth factors can promote chondrocyte proliferation, expanding the population of these cells critical for cartilage repair [145]. During cell growth, differentiation, and immune responses, cytokines, including IL-1β and TGF-β, stimulate and regulate chondrocyte proliferation, activation, and function, playing a crucial role in maintaining and repairing cartilage tissue [53]. To induce phenotypic polarization of macrophages associated with constructive cartilage remodeling, Tian et al. [146] studied two doses of IL-4 to explore the effect of accurate immunomodulation on decellularized cartilage ECM scaffolds for cartilage regeneration. The in vivo data indicate that optimal M1/M2 macrophage signature is achieved through proper IL-4 delivery, thus creating a microenvironment that promotes cartilage healing and regeneration. Additionally, Wu et al. [147] co-encapsulated the chemokine of stromal cell-derived factor-1 (SDF-1) and chondrogenic induction molecule (kartogenin, KGN) in homogeneous and monodisperse PLGA microspheres via microfluidic technology. These microspheres, which can be sustainably released with matching synergistic effects, were incorporated into hyaluronic acid injectable scaffolds to repair full-thickness articular cartilage defects in rabbits. The regenerated tissue exhibited good cartilage tissue characteristics and was well integrated with the surrounding tissue.

#### 4.1.2 Stimulation of stem cells proliferation

Bioactive factors stimulate stem cell differentiation into chondrocytes through signaling pathways, matrix alterations, and gene regulation [148]. Loaded onto suitable biomaterials, these bioactive molecules effectively promote spontaneous healing [149]. Anhydroicaritin (AHI), a small herbal molecule, has been identified as a bioactive factor promoting articular cartilage stem cells (ACSCs) differentiation. Cui et al.131 designed an injectable nanocomposite platform to enhance cartilage regeneration, integrating chitosan hydrogels, joint ACSCs, mesoporous SiO_2_ nanoparticles (mSiO_2_ NPs), and AHI. The synergistic action of mSiO_2_ NPs and pore channels in the hydrogel shows an excellent sustained release effect (Fig. 9B) [126]. Polypeptides are short-chain proteins composed of amino acids, typically 2–50 amino acid residues [150]. Polypeptide-based drugs with strong bioactivity can modulate the extracellular matrix environment, growth factor release, and signaling pathways, thereby facilitating the regeneration and differentiation of chondrocytes [103]. Although IGF-1 induces ADSCs to differentiate into chondrocytes while treating injured cartilage, it is prone to short tissue retention and instability. To address this, Nap-FFG-GYGSSSRRAPQT, a supramolecular peptide with superior properties to IGF-1, can replace it as a tissue engineering therapy for inducing ADSC differentiation into chondrocytes [151]. The β-folded secondary structure of Nap-FFG-GYGSSSRRAPQT is responsible for superior properties, and conformation transformation is expected to be applied in other tissue engineering therapies [152]. However, challenges remain in treating cartilage disease with polypeptide materials, such as ensuring their stability, improving their delivery efficiency, and overcoming their metabolism and clearance. Martin et al. [153] developed a cell-free electrospun fibrous HA scaffold that provides factors to enhance cartilage repair, namely SDF-1α and TGF-β3. SDF increases the recruitment and infiltration of MSCs, while TGF enhances cartilage tissue formation [154]. Chen et al. [155] designed a biodegradable, nontoxic DN hydrogel of PEG and KGN-conjugated chitosan to sustainably and efficiently activate multiple cartilage-related genes and signaling pathways to maintain the survival and chondrogenic differentiation of peripheral blood-derived MSCs. Mao et al. [156] developed a functional acellular silk fibroin methacryloyl-hyaluronic acid methacryloyl scaffold for cartilage repair, which can release growth factors E7 and TGF-β1 in sequence. Although research on cytokine therapy for cartilage is still in the early stages, more clinical trials are needed to verify safety and efficacy.

#### 4.1.3 Synthesis of cartilage matrix

Bioactive factors can promote the synthesis of cartilage matrix, thereby enhancing the mechanical properties and biological functions of cartilage [159]. Biomaterial-guided gene therapy can improve cartilage repair by precisely controlling the spatiotemporal release of treatment sequences [160]. Based on molecular biology techniques, gene recombination therapy transfer the gene with a therapeutic effect into the human body through recombination technology [62]. Maihofer et al. [161] used the rAAV vector on ALG saline gel to encode human IGF-I to achieve full-layer cartilage repair. Compared to a control group of miniature pigs (lacZ/AlgPH155), persistent IGF-I overexpression in IGF-I/AlgPH155 significantly repaired tissue defects after one year of microfracture treatment. IGF-I/AlgPH155 significantly improved cartilage repair parameters (cell density, semi-quantitative total histological score, matrix deposition) within one year without adverse or immune responses. rAAV gene transfer in biomaterials is a valuable clinical method to promote cartilage repair [90].

#### 4.1.4 Inhibition of chondrocyte apoptosis

Chondrocyte apoptosis inhibition can promote chondrocyte proliferation and increase cartilage tissue matrix synthesis [165]. miRNA therapy is an emerging therapeutic approach for treating cartilage damage by modulating cellular gene expression by manipulating specific miRNA molecules [166]. miRNA can effectively regulate gene expression by binding to the mRNA of target genes, inducing degradation or inhibiting translation into proteins. The decreased expression of miR-17 in osteoarthritis chondrocytes contributes to cartilage aging. Sivaraj et al. [158] demonstrated that the supplementation of exogenous miR-17 and promotion of endogenous growth differentiation factor 5 expression effectively targeted pathological catabolic factors such as aggrecanase-2, MMP3/13, and nitric oxide synthase-2 (NOS2). Single-cell RNA sequencing analysis of the cartilage revealed two distinct populations of superficial chondrocytes (C1/C2). C1 expresses catabolic factors, including MMP2, while C2 has synovial features. Furthermore, miR-17 may help maintain physiological anabolic and catabolic balance by decreasing HIF-1α signaling [162] (Fig. 9C). The study showed that miR-17 could maintain cartilage homeostasis and prevent cartilage aging [167]. Thus, through intervention in miRNA expression, regulation of chondrocyte differentiation and metabolism can be achieved, promoting chondrocyte regeneration and repair. Cartilage repair processes can be compromised due to aberrant receptor expression. For instance, prostaglandin E receptor 4 (EP4) expression significantly increases after articular cartilage injury. In a mouse model of cartilage defects induced by microfracture surgery, Jin et al. [163] found that specific loss of EP4 accelerated cartilage anabolism and inhibited cartilage catabolism. EP4 enhances the formation of mature articular cartilage rather than fibrocartilage, and reduces joint pain. The EP4 antagonist HL-43 can promote chondrocyte differentiation and anabolism with ideal biocompatibility. HL-43 promotes extracellular chondrocyte matrix generation and cell differentiation, which inhibits the degradation of the human articular cartilage matrix. HL-43/EP4 changes cartilage anabolic metabolism by regulating cAMP/PKA/CREB/Sox9 signals at the molecular level. Therefore, receptor antagonists can effectively mediate physiological or pathological processes by binding to receptors on the chondrocyte surface, preventing receptor activation, or reducing the activity of the receptor [168].

### 4.2 Stem cells

Stem cells are self-renewing cells capable of generating multiple differentiated progeny [169]. The transplantation of stem cells is an emerging approach for repairing cartilage damage, whereby stem cells are transplanted to the injury site to promote regeneration and repair [143,170]. Through the secretion of growth factors and cytokines, stem cells have the potential to enhance chondrocyte proliferation and differentiation, thereby facilitating the regenerative and reparative processes of cartilage tissue [143]. Furthermore, stem cells can promote the generation and accumulation of cartilage matrix by secreting various matrix proteins and bone matrix poietin. Stem cells can differentiate into chondrocytes, cartilage matrix, and ectodermal cartilage cells, thereby providing a means for direct replacement of damaged cartilage tissue [171]. While technical challenges and safety concerns remain with stem cell transplantation, it is expected to become a crucial therapeutic approach for cartilage damage as technology advances.

Human umbilical cord blood-derived MSCs (HUCB-MSCs) refer to MSCs isolated from the blood of human umbilical cords. The recipient's immune system rarely rejects them compared to other sources of stem cells, such as bone marrow-derived MSCs [172]. Huang et al. [173] designed an enzyme-crosslinked gelatin/hydroxyapatite (HAP) hybrid material as a scaffold for HUCB-MSCs, produced by micro-extrusion 3D bioprinting. HAP doping can improve the rheology and gel dynamics of the composites and better support HUCB-MSCs adhesion, growth, and proliferation. However, enabling stem cells at the injury site requires a highly biocompatible scaffold to control porosity and geometry precisely. The differentiation of stem cells at the damaged site of articular cartilage in the HAP scaffold remains to be further explored.

**Fig. 10:**
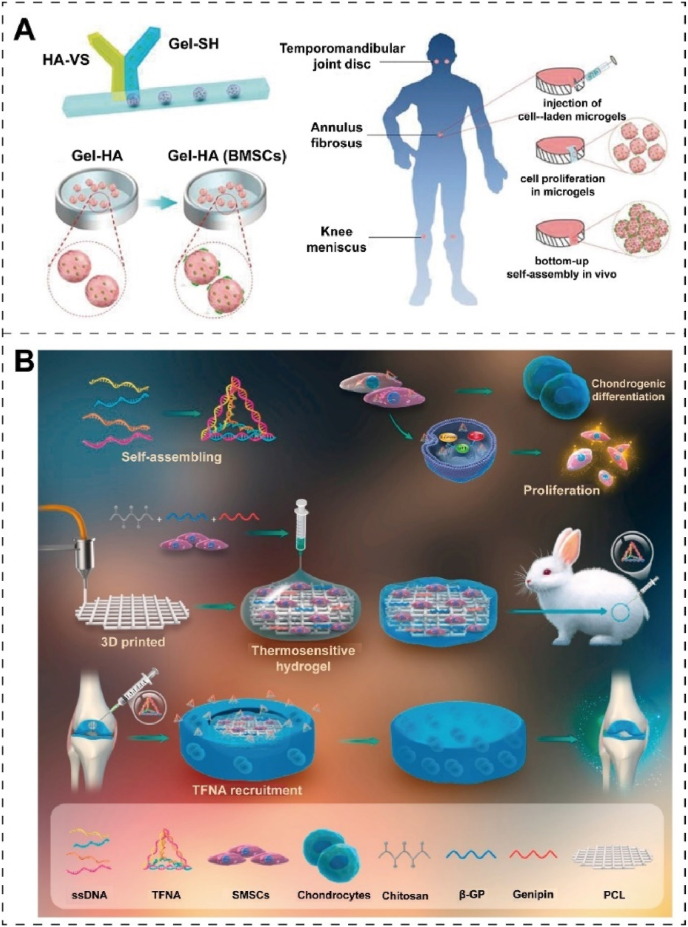
(A) Schematic representation of cartilage regeneration by Gel-HA microgel containing BMSCs: A BMSCs-containing Gel-HA microgel was generated by thiol-Michael addition reaction microfluidic between HA-VS and Gel-SH; In vitro culture of microgels containing BMSCs; Injection and in vivo self-assembly of microgels containing BMSCs. Reproduced with permission [176]. Copyright 2019, Wiley-VCH. (B) Schematic representation of cartilage defects repaired by a hybrid chitosan hydrogel/3D printed PCL scaffold containing SMSCs promoted by TFNA in rabbits. Reproduced with permission [178]. Copyright 2019, Elsevier.

BMSCs are adult stem cells found in the bone marrow, which can differentiate into various types of cells, such as the cells of bone, cartilage, and fat [174]. The in situ cartilage repair by integrating endogenous BMSCs and appropriate bioactive materials has attracted extensive attention [175]. This strategy first recruits enough endogenous stem cells to the injured area by constructing a bioactive scaffold [170]. Then it promotes cartilage differentiation of MSCs to achieve cartilage in situ regeneration and repair, giving full play to the role of autologous MSCs and avoiding problems related to cell operation. Cao et al. [176] proposed a new preparation method of a microgel containing stem cells, which could be injected into cartilage defects in a minimally invasive manner. Thiolated gelatin (Gel-SH) and sulfonated vinyl HA (HA-VS) are first synthesized and mixed. The mixture was loaded with BMSCs by a drop-based microfluidic method and then gelated by a thiol-Michael addition reaction. The injection of Gel-HA microgel containing BMSCs and its self-assembly can regenerate cartilage defects, providing an efficient cartilage regeneration tissue engineering method (Fig. 10A). HA can bind to the CD44 receptor of chondrocytes and promote phenotype maintenance and chondrogenesis as an essential component of the cartilage matrix. Wei et al. [177] constructed injectable HA hydrogel loaded with BMSCs spheres and short fiber fillers for the noninvasive repair of cartilage defects. F_K_-CS comprises cell spheres and short fibers loaded with KGN, which serves as a matrix to promote cell growth and regulate cell differentiation. When injected into the hydrogel, F_k_-CS up-regulated the expression of aggrecan, col II and Sox9. In the osteochondral defect model established by surgery, the injection of the hydrogel can repair the integrity of the tissue and promote the normalization of chondrocytes morphology and ECM deposition.

As a tissue-specific stem cell, synovial mesenchymal stem cells (SMSCs) exhibit low immunogenicity, high proliferation rate and excellent chondrogenic differentiation potential. Li et al. [178] developed a hybrid chitosan hydrogel and 3D PCL scaffold containing tetrahedral framework nucleic acid (TFNA) and SMSCs for cartilage regeneration. The PCL scaffold provides mechanical force support, while TFNA is a DNA nanomaterial that can regulate various cell biological behaviors. Chitosan with a positively charged glucosamine group can bind to negatively charged DNA by electrostatic interaction and recruit free TFNA after injection into the joint space. TFNA promoted the proliferation of SMSCs and cartilage regeneration and differentiation by improving the regeneration microenvironment (Fig. 10B). This study highlighted the targeted recruitment of TFNA by electrostatic interaction to exert their effects on stem cells and pointed out the more significant therapeutic potential of TFNA in cartilage repair species. Mesenchymal progenitor cells (MPCs) play a role in maintaining and repairing cartilage [179], and Krawetz et al. [180] found that synovial MPCs secretes aggrecan in healthy joints. The absence of alpha-2 macroglobulin (A2M) inhibits aggrecan secretion in the synovial MPCs of OA, and overexpression of A2M causes aggrecan to be secreted normally. Intraarticular injection of aggrecan inhibits cartilage degeneration and stimulates cartilage repair in a mouse model of osteoarthritis.

### 4.3 EVs

EVs are nanoscale membrane-bound carriers that all cells can secrete, containing specific biomolecules such as miRNA, mRNA, and proteins [181,182]. As crucial mediators of intercellular communication, EVs transfer these biomolecules to cartilage cells, modulating their gene expression and cellular functions [154]. Moreover, EVs can transmit antigenic information to immune cells and carry growth factors and cytokines that affect chondrocyte proliferation and growth [183]. Furthermore, EVs regulate the expression of signaling molecules involved in cartilage repair, promoting cartilage's therapeutic biological activities [184]. The underlying mechanisms of the cell-origin effect of EVs on cartilage regeneration are complex and multifactorial [183]. Selecting the appropriate EVs source is critical for their therapeutic use in tissue regeneration.

#### 4.3.1 EVs of stem cells

**Fig. 11:**
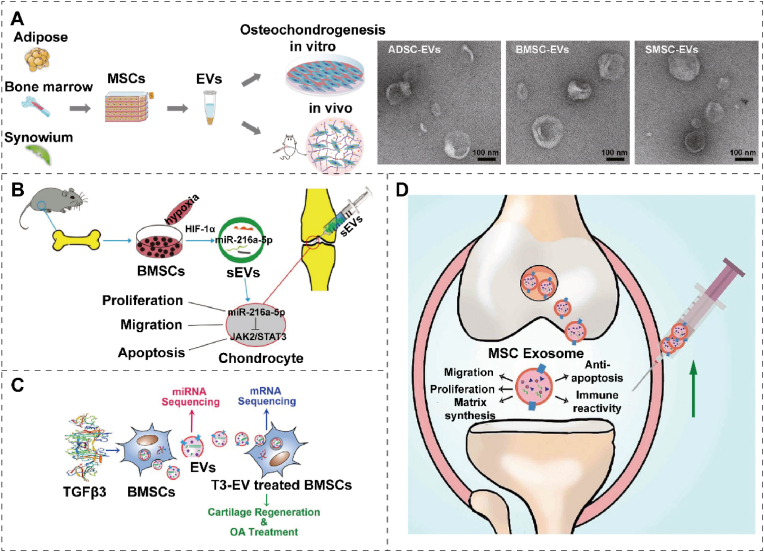
Mechanism of EVs derived from different cells on cartilage repair. (A) Schematic preparing steps and TEM image of ADSC-EVs, BMSC-EVs, and SMSC-EVs for cartilage regeneration in vitro and in vivo. Reproduced with permission [185]. Copyright 2021, Elsevier. (B) Schematic representation of sEVs secreted by BMSCs induced by HIF-1α injection to promote JAK2/STAT3 signaling pathway in miR-216a-5p for cartilage repair. Reproduced with permission [188]. Copyright 2021, Elsevier. (C) Schematic representation of EVs secreted by BMSCs pretreated with TGF-β3 to promote cartilage regeneration. Reproduced with permission [189]. Copyright 2022, Springer Nature. (D) Schematic diagram of the mechanism of action of MSCs exosomes in cartilage repair. MSCs-mediated exosomes enhance cell proliferation, migration, and matrix synthesis, reduce cell apoptosis, and regulate immune responses in cartilage regeneration. Reproduced with permission [190]. Copyright 2018, Elsevier.

Implanting MSCs directly for cartilage tissue engineering is difficult due to immune rejection and cellular viability loss [175]. However, EVs of MSCs have the potential to overcome these problems because of molecular lipid membranes. Therefore, it is essential to examine how the tissue origin of MSCs affects the therapeutic potential of the corresponding EVs for cartilage regeneration. Li et al. [185] cultured rat MSCs isolated from adipose, bone marrow, and synovium to obtain their corresponding EVs (ADSC-EVs, BMSC-EVs, and SMSC-EVs, respectively). ADSC-EVs were more effective than BMSC-EVs or SMSC-EVs in promoting migration, proliferation, chondrogenic differentiation of BMSCs in vitro, and cartilage regeneration in a mouse model [186] (Fig. 11A). Proteomics analysis revealed that the tissue origin of the MSCs contributed to distinct protein profiles among the three types of EVs, which potentially induce cartilage and bone regeneration by regulating various signaling pathways such as focal adhesion, ECM-receptor interaction, actin cytoskeleton, cAMP, and PI3K-Akt signaling pathways [187]. MSCs-produced exosomes can co-repair and regenerate osteochondral defects through various reactions, including enhancing cell proliferation and matrix synthesis, reducing cell apoptosis, and modulating immune responses.

The HUCB-MSCs EVs contain a variety of neurotrophic and growth factors that promote neuronal cell growth and regeneration, as well as antioxidant and anti-inflammatory properties [191]. Small extracellular vesicles (sEVs) can be washed away and degraded in vivo without protection [192]. Hu et al. [193] also explored the mechanism of HUCB-MSCs-sEVs in promoting cartilage regeneration. In vitro experiments show that HUCB-MSCs-sEVs can enhance the migration, proliferation, and differentiation of chondrocytes caused by transferring miR-23a-3p to suppress the level of PTEN and increase the expression of AKT. Pluripotent stem cell-derived EVs have a variety of functions similar to ESCs-derived EVs. Meanwhile, they also express cell surface markers such as CD44, CD73, and CD90, which identify and interact with recipient cells [169]. miRNA in EVs can be transported into other cells, entering the cell and affecting the expression of target genes. Rong et al. [188] demonstrated that the sEVs of BMSCs under hypoxia had a better therapeutic effect on OA cartilage repair than BMSCs under normal oxygen. In sEVs, miR-216a-5p has the function of targeting chondrocytes in the process of OA cartilage repair, and down-regulates the expression of JAK2, the target gene of sEVs miR-216a-5p. HIF-1α induces sEVs secretion from BMSCs in hypoxia, and promotes chondrocyte proliferation, migration, and apoptosis restriction through the JAK2/STAT3 signaling pathway in miR-216a-5p (Fig. 11B). Therefore, hypoxia pretreatment is an effective method to optimize the therapeutic effect of BMSCs-derived sEVs. When pretreated with TGF-β3, EVs secreted by BMSCs possess abundant cartilage-specific miRNAs, which can act on native cells and ultimately enhance chondrogenesis [189]. In addition, miR-455, one of the most enriched miRNAs in EVs, targets SOX11 and regulates the downstream FOXO signaling pathway. The designed T3-EV hydrogel also showed a significant therapeutic effect in the treatment of cartilage defects (Fig. 11C). This research approach applied engineered EVs from chondrogenic primed-BMSCs to cartilage repair and deepened the understanding of EV-miRNA-regulated chondrogenesis in vivo.

#### 4.3.2 EVs of immune cells

EVs released by immune cells, including dendritic cells and macrophages, play a critical role in immune responses, functioning as crucial regulators of intercellular communication and modulators of immune cell activity. These EVs act as messenger molecules to transmit information and modulate immune cell interactions, thereby regulating the duration and intensity of immune responses, and offering potential therapeutic applications in immunomodulatory therapy [194]. Microvesicles (MVs) are a novel intercellular communication mechanism that transfers cellular lipid and protein components to target cells [195]. Neutrophils protect cartilage from damage by sending MVs to perform tasks in tissues they cannot access [196]. To determine the therapeutic function of MVs, Headland et al. [197] examined the role of immune cell-derived MVs in human primary chondrocytes and rodent models. In vitro, MVs can protect cartilage by reducing the adaptive stress homeostasis mediators interleukin 8 and prostaglandin E2 and promoting the expression of anabolic genes in chondrocytes. The injection of a combination of MVs and AnxA1 into the articular cavity in vivo alleviates cartilage degeneration caused by inflammation. Cartilage protection is based on the interaction of the MVs-related AnxA1 with receptor FPR2 (formyl peptide receptor 2)/ALX to increase the production of TGF-β by chondrocytes. Both normal ADSC-derived sEVs (AEs) and inflammation-stimulated ADSC-derived sEVs (IAEs) promote cell proliferation, while IAEs significantly improve cell migration [53]. The study found that IAEs can facilitate M2 macrophage differentiation, potentially through regulating macrophage colony-stimulating factor-1 by high miR-27b-3p expression levels, as revealed by RNA-sequencing analysis. Furthermore, in a rabbit temporomandibular joint (TMJ) condylar osteochondral defect model, both AEs and IAEs promoted TMJ regeneration, with IAEs showing the most significant therapeutic effect. Thus, the study concludes that exposing MSCs to an inflammatory environment can enhance the functions of sEVs, and modified sEVs have improved therapeutic efficacy [198]. Exosomes repair cartilage defects by increasing cell proliferation and accelerating regenerative immunophenotype and matrix synthesis. Adenosine activation of ERK and AKT signaling mediated by exosome CD73 leads to rapid cell proliferation. Exosome treatment showed a regenerative immunophenotype. Specifically, CD163^+^ regenerative M2 macrophages were more infiltrated than CD86^+^ M1 macrophages, accompanied by decreased TNF-α and IL-1β in pro-inflammatory cytokines [190] (Fig. 11D).

#### 4.3.3 EVs of chondrocytes

The potential for human articular chondrocytes (HACs) derived from EVs to promote cartilage regeneration has not been thoroughly investigated. Casanova et al. [199] developed a system for immobilizing EVs selectively present in a conditioned medium from cultures of HACs. An electrospun nanofibrous mesh was activated and functionalized with an anti-CD63 antibody. The chondrogenic potential of the bound EVs was evaluated by culturing human BMSCs (HBMSCs) under basal conditions. EVs derived from HACs induced a chondrogenic phenotype in HBMSCs, characterized by the synthesis of matrix glycosaminoglycans and marked induction of SOX9, COMP, Aggrecan, and Col II. This approach outperformed currently used chondroinductive strategies. Therefore, naturally secreted EVs can promote the chondrogenic commitment of HBMSCs based on medium supplementation without other chemical or genetic chondrogenic inducers. Because of their natural delivery mechanism to cells, targeting ability, stability in biological fluids, and long-distance travel, EVs have great potential for therapeutic applications in various disease areas and drug delivery [200]. Harnessing their targeting ability could provide a wide range of therapeutic opportunities. Thomas et al. [201] explored the impact of delivering WNT3a into large cartilage defects using exosomes as a delivery method. WNT3a successfully assembled on exosomes and triggered WNT signaling in vitro. In vivo, after a single administration of WNT3a-loaded exosomes, canonical WNT signaling was activated for at least one week, leading to improved repair of osteochondral defects even after 8 weeks. WNT3a loaded on exosomes was efficiently delivered into cartilage and facilitated the healing of osteochondral defects [202].

### 4.4 Cartilage organoids

Organoids are 3D cell mass structures formed through the proliferation and differentiation of embryonic or adult stem cells in vitro, exhibiting specific morphological structures and functions [203]. While organoids do not fully recapitulate the complexity of real human organs, they mimic some structural and functional aspects of their model organs [204]. Cartilage organoids have been shown to better represent the cellular state of injured cartilage, allowing for the examination of relevant gene or protein expression during cartilage injury [205]. As such, they provide a valuable model for investigating the molecular mechanisms underlying cartilage injury and offer a convenient platform for drug screening and other therapeutic approaches. Despite their potential, there is currently a shortage of research on cartilage organoids, suggesting a need for further development.

**Fig. 12:**
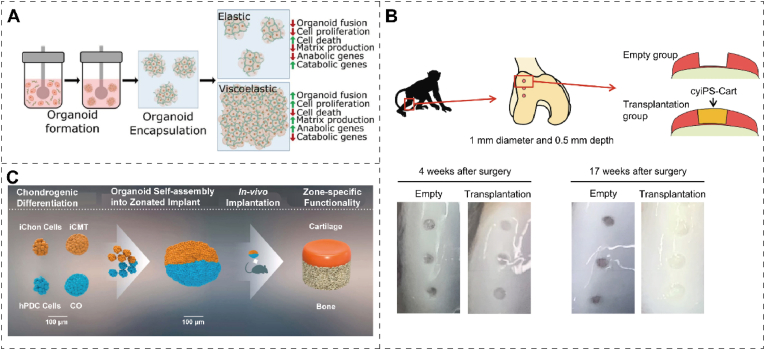
Current advances of cartilage organoids for treating injured cartilage. (A) Schematic of cartilage organoid formation and the effect of hydrogel elasticity and viscoelasticity on tissue in organoid fusion, cell proliferation, cell death, matrix production, anabolic genes, and catabolic genes. Reproduced with permission [206]. Copyright 2021, Elsevier. (B) The diagram shows how building blocks are assembled into zonal constructs. The first step involves creating a hierarchical micromodule implant of chondrocytes derived from human periosteum-derived cells (HPDCs) and induced pluripotent stem cells (IPSCs). The second step shows a diagrammatic representation of how building blocks are assembled into tri-layered constructs. Reproduced with permission [207]. Copyright 2021, Elsevier. (C) Schematic illustration of created chondral defects in the femoral trochlear ridge of the right knee joints of cynomolgus monkeys to establish a primate model for CyiPS-Cart transplantation. The transplanted either CyiPS-Cart or nothing into the defects and observed the gross appearance of the joint surface 4 weeks and 17 weeks after surgery. Reproduced with permission [208]. Copyright 2023, Springer Nature.

As a novel biological tissue engineering method, cartilaginous organoids have great potential in treating injured cartilage. Crispim et al. [206] designed a suspension expansion method to rapidly produce cartilage organoids through bovine chondrocytes in a dynamic culture system. This new method uses a notochordal cell-derived matrix to supplement the culture medium, resulting in cell proliferation, high vitality, and self-assembly with classified organs of the ECM group. These organs are similar to the natural hyaline cartilage, composed of glycosaminoglycans, col II, and type VI collagen. Organoids have Sox9-positive cells embedded in the cell lacuna and the matrix between membranes. In order to produce large-scale tissues, organoids are encapsulated into alginate saline gel with different viscoelasticity. Elastic hydrogels inhibit the growth and tissue formation of organoids. On the contrary, viscoelastic hydrogels allow organoid growth and fusion to form homogeneous tissue with strong mechanical stability, rich in col II and glycosaminoglycans. The 3D culture system is also superior to the traditional 2D method of expanding chondrocytes by wrapping organoids to produce new cartilage in vitro (Fig. 12A). There is limited research on the expansion of chondrocytes, the formation of organoids, and the assembly of new hyaline cartilage. To advance the field, future efforts should prioritize optimizing the formation of cartilage organoids derived from human chondrocytes and investigating the potential therapeutic applications of these human cartilage organoid derivatives for treating cartilage-related conditions.

Bone defects could be repaired using callus organoids derived from human pluripotent stem cells (HPSCs) [209]. After the mesoderm is induced, HPSCs assemble and differentiate into glycosaminoglycan-rich nodules. These nodules gradually mature into cartilage aggregates rich in col II and safranin. After ectopic implantation, the aggregates exhibited the tissue features of hyaline cartilage. Cartilage nodules mature and become mineralized in response to targeted stimulation of growth differentiation. These findings indicate that HPSCs can generate soft callus-like tissue and promote bone healing in vivo [210]. HPSCs hold promise for allogeneic cartilage transplantation as a potential treatment for articular cartilage defects. Nonetheless, the feasibility of allogeneic cartilage transplantation in primate models remains uncertain. In a primate model of chondral defects in the knee joints, Tsumaki et al. [208] demonstrated that allogeneic IPSC-derived cartilage organoids can survive and integrate effectively and remodel articular cartilage (Fig. 12B). After histological analysis, it was discovered that allogeneic IPSC-derived cartilage organoids did not trigger an immune response and were effective in repairing tissue for a minimum of four months. Furthermore, these cartilage organoids could blend in with the host's native articular cartilage and prevent the degeneration of surrounding cartilage. Single-cell RNA sequencing analysis showed that the IPSC-derived cartilage organoids expressed PRG4, which is vital for joint lubrication. As suggested by pathway analysis, it was determined that SIK3 inactivation may play a significant role. Based on these findings, allogeneic transplantation of IPSC-derived cartilage organoids could be a feasible approach to treating chondral defects of the articular cartilage in patients. However, further study is required to determine long-term functional recovery after load-bearing injuries.

From the tissue engineering perspective, organoids can potentially address the medical need to treat deep osteochondral defects [211,212]. However, current strategies struggle to create patterned constructs with distinct biological functions. Papantoniou et al. [207] proposed a modular approach inspired by development, using cartilaginous organoids as building blocks. They created a hierarchical construct consisting of three layers of cartilaginous tissue intermediates derived from HPDCs, representing early, mature, and pre-hypertrophic phenotypes. Subcutaneous implantation in mice resulted in a hybrid tissue, but the non-mineralized part was represented by a collagen type I positive fibrocartilage-like tissue. They generated iPSC-derived cartilage microtissues to create a more stable articular cartilage part. After implanting assembled cartilage microtissues derived from IPSCs subcutaneously, a uniformly cartilaginous tissue that tested positive for col II but negative for osteocalcin was observed. Finally, the researchers created dual tissues with a pre-engineered zonal pattern by combining IPSC-derived cartilage microtissues and pre-hypertrophic cartilage organoids. When subcutaneously implanted, these tissues reflect the intended zonal pattern, exhibiting both cartilaginous and bony regions. (Fig. 12C). The assembly of functional blocks presents new possibilities for creating composite tissue-engineered implantations with pre-programmed living building blocks that embed zone-specific functionality. Chondral organoids hold promising potential for the regeneration and replacement of articular cartilage [213]. However, to actualize this prospective therapeutic direction, in-depth exploration of critical factors in organoid cultivation is imperative. This encompasses the quest for materials exhibiting enhanced biocompatibility and biodegradability to mitigate foreign body reactions and promote cell adhesion, proliferation, and differentiation post-implantation [214]. Furthermore, the optimization of growth factors and bioactive molecules essential for cartilage regeneration represents a pivotal research avenue. Precise modulation of these factors is crucial for effectively guiding the growth and differentiation behavior of organoids [215]. Additionally, there is a need for further investigation into the matrix material conditions of organoid cultivation to ensure the cultivation of transplantable tissues possessing native cartilage characteristics, facilitating their widespread clinical applicability.

## 5 Composite bioactive scaffolds in clinical practice

**Fig. 13:**
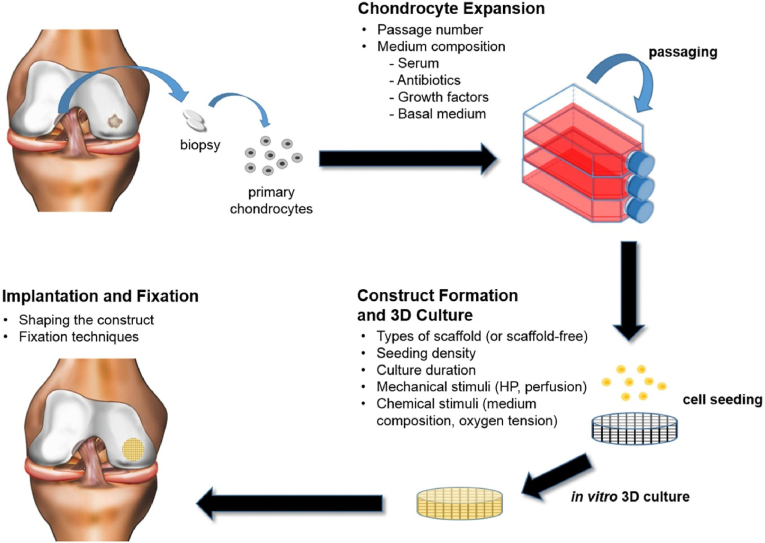
Schematic diagram a quintessential tissue engineering paradigm for crafting cartilage products under scrutiny. Each stage, as outlined, allows for the manipulation of various factors. Reproduced with permission [216]. Copyright 2016, Elsevier.

Current goals in cartilage regeneration extend beyond simple tissue formation, focusing on achieving cartilage regeneration under abnormal conditions to mimic clinical scenarios. Treatment requirements vary depending on the location of the injury or primary disease condition. Numerous materials have undergone clinical translation with advancements in regenerative medicine and tissue engineering (Fig. 13). Products for repairing cartilage and osteochondral defects available or soon to be marketed can be categorized into two types (1) allogeneic grafts (e.g., BioSeed®-C189, BioCart™ II190, BST-Cargel®191, CaReS®192, NOVOCART®3D193, INSTRUCT194, Hyalograft C195): and (2) acellular scaffolds (Agili-CTM196, Cartiva® SCI197, Chondro-Gide®198) (Table 3).

**Table 3:** Commercial products of composite scaffold for articular cartilage repair.

Product	Type	Scaffold composition	Company	Ref.
MACI	Autologous chondrocytes	Porcine collagen membrane	Vericel Corporation	
BioSeed®-C	Autologous chondrocytes	Polylactic acid/polydioxanone wool fabric	BioTissue Technologies GmbH	[217]
BioCart™ II	Autologous chondrocytes	Fibrinogen/hyaluronic acid scaffold	Histogenics	[219]
BST-Cargel®	Autologous peripheral whole blood	Mixed solution of chitosan and glycerophosphate buffer	Smith & Nephew	[220]
CaReS®	Autologous chondrocytes	Type I collagen hydrogel	Arthro Kinetics Biotechnology	[218]
NOVOCART®3D	Autologous chondrocytes	Biphasic type I collagen scaffold	TETEC	[221]
INSTRUCT	Primary autologous chondrocytes and bone marrow cells	Poly (ethylene oxide ethyl terephthalate)/poly (butanediol terephthalate)	CellCoTec	[222]
Hyalograft C	Autologous chondrocytes	Esterified hyaluronic acid	Anika Therapeutics	[223]
Agili-CTM	Cell-free implants	Natural inorganic calcium carbonate biphasic scaffold	CartiHeal	[224]
Cartiva® SCI	Cell-free implants	Polyvinyl alcohol cryogel	Stryker	[48]
Chondro-Gide®	Cell-free implants	Bilayer type I/III collagen membranes	Geistlich	[225]

Different cartilage injuries necessitate tailored treatments, highlighting the need for personalized patient therapies. These newly approved products offer surgeons a novel treatment option. MACI, developed by Vericel Corporation headquartered in Cambridge, Massachusetts, USA, is one such innovation. The U.S. Food and Drug Administration has granted approval for MACI, the first tissue-engineered product for knee cartilage injury treatment, cultivated on a porcine collagen membrane. Autologous cartilage cells derived from the patient's knee cartilage tissue biopsy serve as the cell source in this product. Researchers culture these cells and implant them onto a purified, absorbable, porcine-derived collagen membrane. MACI is suitable for repairing adult cartilage defects, with each MACI graft consisting of sheets containing 500,000 to 1 million cells/m2. The number of MACI sheets used depends on the area of cartilage damage, ensuring complete coverage. Multiple sheets can be employed for multiple cartilage defects, and each sheet can be trimmed to fit the damaged area. A two-year study involving 144 patients (72 in each group) demonstrated that MACI effectively alleviated pain and improved functionality. All patients treated with MACI succeeded, with many participating in a subsequent three-year follow-up study. Overall efficacy data support the long-term clinical benefits of MACI for cartilage defect patients.

Commercially tailored products are more flexible and can more appropriately supplement the site of cartilage injury. BioSeed®-C facilitates the secure fixation of chondrocyte transplants beneath cartilage, enabling the treatment of defects in partially compromised cartilage edges [217]. This innovation permits customization of graft size, uniform cell distribution, and transplant delivery through arthroscopy or open-joint surgery. The biomechanical incongruity between engineered cartilage implants and adjacent native cartilage poses a significant challenge. While achieving consistent biochemical properties in vitro is achievable, the immature structural development often results in inferior biomechanical characteristics compared to mature articular cartilage. Consequently, cultivating chondrocytes in situ emerges as a strategic approach to enhance the biomechanical integrity of newly formed cartilage. CaReS®, developed by Arthro Kinetics Biotechnology, is a Type I collagen hydrogel encapsulating native autologous chondrocytes [218]. Compared to other products, CaReS® exhibits relatively lower cell density but achieves the required high cellularity for new tissue formation through cell proliferation and migration. After six weeks of in vitro culture, primary chondrocytes within CaReS® gel exhibit a 33-fold expansion.

From the range of cell-free cartilage repair or regeneration commercial products, it's evident that the materials selected predominantly comprise biopolymer polysaccharides, such as collagen and hyaluronic acid. These materials may offer good biocompatibility, controlled degradation rates, and manageable degradation products. The products are typically injectable or membrane-based, enhancing their usability and facilitating cartilage repair. Agili-CTM, developed by CartiHeal, is a cell-free, off-the-shelf implant for cartilage and osteochondral defects in traumatic and osteoarthritic joints [224]. Cartiva®SCI, developed by Stryker, is a novel osteochondral defect repair implant with properties akin to cartilage [48]. It is approved for treating first metatarsophalangeal joint pain and degeneration, with or without mild hallux valgus or post-traumatic arthritis (hallux limitus or hallux rigidus) in patients. The material used is polyvinyl alcohol cryogel, the only FDA-approved synthetic polymer in the category of cartilage-like materials. Chondro-Gide®, developed by Geistlich, is a bilayer Type I/III collagen membrane made from highly refined porcine collagen through a stringent quality assurance process [225]. Its smooth and compact surface prevents the loss of cell diffusion, while the rough, porous lower layer facilitates cell attachment and growth. This product is specifically designed for cartilage regeneration, harnessing the body's innate healing potential for human cartilage regeneration. These products can serve as a valuable foundation for the research and development of improved next-generation products. Furthermore, with the advancement of autologous chondrocyte transplantation and stem cell tissue engineering technologies, combining cells with biocompatible scaffold materials can lead to cost-effective cartilage regeneration products to meet the demands of cartilage defect repair.

## 6 Conclusion and future perspectives

Cartilage is an avascular and aneural connective tissue with limited regenerative capacity, necessitating external intervention to promote repair after injury. Given the challenges associated with repairing cartilage injuries, this review summarizes various biomaterial-based strategies for cartilage repair. A noteworthy advancement in this realm is the development of stimuli-responsive smart scaffolds, capable of precisely detecting and addressing cartilage damage through targeted responses to physical cues. These emerging scaffolds, exemplified by 3D-printed and cartilage bionic scaffolds, provide robust mechanical support for chondrocytes and the extracellular matrix. Biologically active agents play a pivotal role in cartilage repair, primarily by promoting chondrocyte proliferation and differentiation by utilizing bioactive factors, stem cells, EVs, and organoids. Additionally, various commercial products have been introduced to address cartilage injuries. This review delves into the mechanisms underpinning cartilage injury repair and underscores their potential clinical implications.

Despite challenges in clinical translation and practical application, the field of cartilage repair materials holds great promise for providing innovative solutions to the treatment of cartilage injuries. Nevertheless, several critical issues must still be addressed to improve the effectiveness and feasibility of these materials.

(1) Excellent biocompatibility reduces rejection reactions and reduces tissue damage. However, some materials still have issues with poor biocompatibility, leading to inflammation after implantation. Cartilage repair materials should also quickly and effectively bond with surrounding tissues to promote the repair process. Therefore, improving the biocompatibility of cartilage repair materials and optimizing their bonding capabilities with surrounding tissues remain essential goals in this field.
(2) While many animal experiments and preliminary human trials have demonstrated the potential of stem cells in treating cartilage injuries, further research is necessary to optimize this therapeutic approach fully. Specifically, the optimal stem cell source, ideal treatment duration, and optimal transplantation modality require further investigation. Moreover, the long-term effects and potential risks of stem cell treatment for cartilage injuries also necessitate additional research. Therefore, future studies should address these critical issues to maximize the potential of stem cell therapy for treating cartilage injuries.
(3) Using EVs for cartilage bioremediation shows promise, but specific protocols for their utilization require further exploration and optimization. Critical areas of investigation include accessing EVs, selecting appropriate carriers and biomaterials, and controlling vesicle release and targeted delivery. Therefore, future studies should address these challenges to optimize the therapeutic potential of EVs for cartilage bioremediation.
(4) Cartilaginous organoids as a tissue model composed of biomaterials and cells. Many areas deserve studying, including disease modeling, drug discovery, and regenerative medicine. Future studies should address these critical areas, including developing optimal cell sources and culture conditions, and enhancing functional and phenotypic characterization of the organoids.
(5) Improving the machinability of the materials and reducing their cost is crucial to making cartilage repair materials more practical for industrial and clinical applications. High machinability would enable more efficient manufacturing processes while reducing the cost of these materials, making them more accessible to a larger patient population. These are essential focus areas for researchers and developers working on cartilage repair materials.

## CRediT authorship contribution statement

**Mingkai Wang:** Conceptualization, Data curation, Formal analysis, Investigation, Methodology, Project administration, Resources, Software, Supervision, Validation, Visualization, Writing - original draft, Writing - review & editing. **Yan Wu:** Conceptualization, Data curation, Formal analysis, Investigation, Resources, Validation. **Guangfeng Li:** Conceptualization, Formal analysis, Software, Supervision. **Qiushui Lin:** Conceptualization, Formal analysis, Methodology, Project administration, Supervision. **Wencai Zhang:** Conceptualization, Investigation, Resources, Supervision, Validation, Visualization. **Han Liu:** Data curation, Investigation, Project administration, Resources, Supervision, Validation, Writing - review & editing. **Jiacan Su:** Conceptualization, Data curation, Formal analysis, Funding acquisition, Methodology, Project administration, Resources, Supervision, Validation, Writing - review & editing.

## Declaration of competing interest

The authors declare that they have no known competing financial interests or personal relationships that could have appeared to influence the work reported in this paper.

## Data Availability

No data was used for the research described in the article.
